# Deciphering failure paths in lithium metal anodes by electrochemical curve fingerprints

**DOI:** 10.1093/nsr/nwaf158

**Published:** 2025-04-24

**Authors:** Zhihong Piao, Zhiyuan Han, Shengyu Tao, Mengtian Zhang, Gongxun Lu, Lin Su, Xinru Wu, Yanze Song, Xiao Xiao, Xuan Zhang, Guangmin Zhou, Hui-Ming Cheng

**Affiliations:** Tsinghua Shenzhen International Graduate School, Tsinghua University, Shenzhen 518055, China; Tsinghua Shenzhen International Graduate School, Tsinghua University, Shenzhen 518055, China; Tsinghua Shenzhen International Graduate School, Tsinghua University, Shenzhen 518055, China; Tsinghua Shenzhen International Graduate School, Tsinghua University, Shenzhen 518055, China; Tsinghua Shenzhen International Graduate School, Tsinghua University, Shenzhen 518055, China; Tsinghua Shenzhen International Graduate School, Tsinghua University, Shenzhen 518055, China; Tsinghua Shenzhen International Graduate School, Tsinghua University, Shenzhen 518055, China; Tsinghua Shenzhen International Graduate School, Tsinghua University, Shenzhen 518055, China; Tsinghua Shenzhen International Graduate School, Tsinghua University, Shenzhen 518055, China; Tsinghua Shenzhen International Graduate School, Tsinghua University, Shenzhen 518055, China; Tsinghua Shenzhen International Graduate School, Tsinghua University, Shenzhen 518055, China; Institute of Technology for Carbon Neutrality, Shenzhen Institute of Advanced Technology, Chinese Academy of Sciences, Shenzhen 518055, China; Faculty of Materials Science and Energy Engineering, Shenzhen University of Advanced Technology, Shenzhen 518055, China; Shenyang National Laboratory for Materials Science, Institute of Metal Research, Chinese Academy of Sciences, Shenyang 110016, China

**Keywords:** lithium metal batteries, lithium metal anodes, failure mechanism, electrochemical curves, machine learning

## Abstract

Understanding anode failure mechanisms in lithium metal batteries (LMBs) is crucial for their use in energy storage, as the anode directly affects battery stability and electrolyte selection. Unfortunately, post-mortem methods reveal failure outcomes but often miss dynamic progressions, obscuring cause-and-effect relationships in failure evolution. Leveraging domain knowledge informed machine learning and a 4-year dataset of over 18 000 cycles and 12 million data points, from cells cycled to failure, we uncovered a correlation between initial lithium plating/stripping behavior and subsequent anode changes, enabling the identification of early indicators for distinct failure types. Our model accurately predicts failure types using only the first two cycles, less than 2% of the data, demonstrating the effectiveness of initial curve features as electrochemical fingerprints. Key electrochemical fingerprints describing lithium microstructure and its interphase with the electrolyte are validated to be critical to kinetics and reversibility degradation. Specifically, the fingerprints influence the formation of ineffective interphase regions (lacking intimate contact with the lithium metal) and inactive lithium, which in turn lengthen charge carrier (lithium-ion and electron) transport paths, leading to poorer kinetics and reversibility. The fingerprints and model generalize well across typical published electrolyte systems with low misidentification, demonstrating versatility and practicality. Broadly, this study using a pre-mortem prediction method deepens understanding of lithium metal anode failure mechanisms by uncovering the root causes of kinetics and reversibility degradation from fingerprints hidden in initial cycles instead of a post-mortem manner, facilitating the rapid assessment of battery reliability and development of electrolytes.

## Teaser

Early-cycle electrochemical curves act as distinctive fingerprints to predict lithium metal anode degradation path, enabling machine learning-driven design of stable lithium metal batteries.

## INTRODUCTION

Lithium metal anodes contribute to superior energy density in batteries compared to traditional graphite anodes used in lithium-ion batteries [1–5]. The performance and longevity of these anodes in lithium metal batteries (LMBs) are significantly influenced by electrolytes, which govern the formation of the solid electrolyte interphase (SEI) and subsequently affect changes in the anode until failure [6–10]. Current electrolyte designs are tailored to specific operational scenarios such as low temperatures [11,12], fast charging [13–15], and high voltages [16–20]. In different scenarios, the anode side faces unique challenges and exhibits varying tolerances for various types of failure, with poorer kinetics and reversibility being the two most critical factors [21,22]. For instance, at a low temperature or under fast-charging conditions, the primary concern is the degradation of electrochemical kinetics. In high-areal-capacity applications, the focus shifts to the anode's ability to accommodate increased lithium-ion deposition, which impacts the reversibility of lithium plating/stripping. A thorough understanding of the mechanisms of these types of failure is essential for the screening and design of high-performance LMB electrolytes that can meet the demands of various applications.

**Figure 1.**
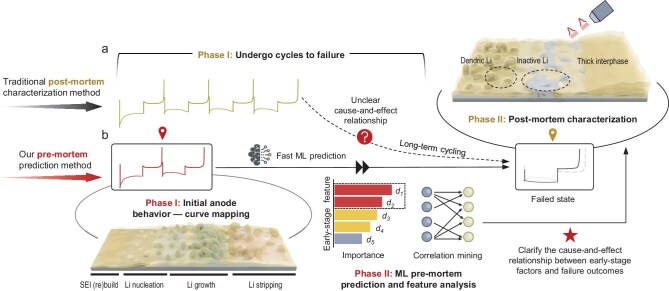
Schematic of (a) the traditional post-mortem characterization method for studying failure mechanisms and (b) the pre-mortem prediction method used in this study. The initial-two-cycle features serve as early-stage electrochemical fingerprints for predicting failure types.

There are many interlinked factors involved in the changes to the lithium metal anode during cycling which makes an understanding of its failure difficult to achieve. Most current research on battery failure mechanisms is based on post-mortem characterizations [21,23–25], which involves assembling batteries, subjecting them to cycling until they fail, and then disassembling them to identify the failure outcomes and potential causes for the failure (Fig. 1a). While this approach provides valuable insights, it cannot identify the dynamic changes that result in failure, obscuring cause-and-effect relationships in failure evolution. Even with operando characterizations, limited data from a small number of samples, restricted to specific electrolyte systems, limits our ability to identify reliable microscopic origins of the failure, and the results of the study may not be universally applicable. Therefore, expanding sample numbers and collecting comprehensive data on a wide range of variables and processes involved in battery evolution is necessary to understand the reasons for failure. This will involve large-scale data-driven analysis.

Electrochemical curves reflect the dynamic electrochemical process, providing a more complete representation of the process than static characterization methods [26,27], while this valuable resource has often been overlooked. Moreover, these data are more realistic, objective, and free from contamination. The complexity and number of such datasets necessitate the use of machine learning (ML) techniques. In contrast to traditional post-mortem analysis, ML enables the establishment of correlations between the early and late stages of the battery cycling process, thereby offering deep insights into the mechanisms causing different types of failure by interpreting the critical features. Furthermore, the high accuracy and generalizability of the ML models not only ensures the validity of the conclusions but also eliminates the need for repeated experiments.

In this study, we collected electrochemical curves of lithium plating/stripping at ambient temperatures with various electrolytes to map lithium behavior at the anode and investigate failure mechanisms through the pre-mortem prediction method (Fig. 1b). The dataset used in this study encompasses a wide range of representative electrolyte types, including low-concentration, weakly-solvated, and (localized) high-concentration electrolytes with various salts, solvents, and additives, covering the majority of mainstream electrolytes in current battery research. We developed a universal classification framework for failure types based on curve evolution patterns, focusing on the kinetics and reversibility degradation of lithium plating/stripping. Using the electrochemical datasets and domain knowledge-informed ML, we discovered the intrinsic correlations between initial cycling stages and types of failure, revealing the cause-and-effect relationship underlying lithium metal anode failure at ambient temperatures. Our failure discrimination model, leveraging data from just the first two cycles—constituting <2% of the total collected data from failed batteries—exhibits strong predictive capabilities with Area Under the Curve (AUC) values of 0.98 and 0.86 for the training and testing datasets, respectively. This means the first two cycles act as the early-stage electrochemical fingerprints, enabling faster pre-mortem prediction of failure types and reducing the time needed for their identification. Through fingerprint analysis, we have identified critical indicators related to the structure of the lithium and its interphase with electrolyte, which determines the way the anode will fail. These characteristics influence the formation of ineffective interphase regions (lacking intimate contact with the lithium metal) and inactive lithium, which greatly affect the transport of charge carriers (both lithium-ions and electrons) in the lithium metal anode, and determine the type of failure. The proposed fingerprints and model developed from a comprehensive dataset are universal and have been validated across several published electrolyte systems covering typical electrolyte types, highlighting their versatility and practicality as useful tools. Our research provides deep insight into the failure mechanisms of lithium metal anodes and facilitates the screening and development of new electrolytes.

## RESULTS

### Electrochemical curves, failure types, and model construction

**Figure 2.**
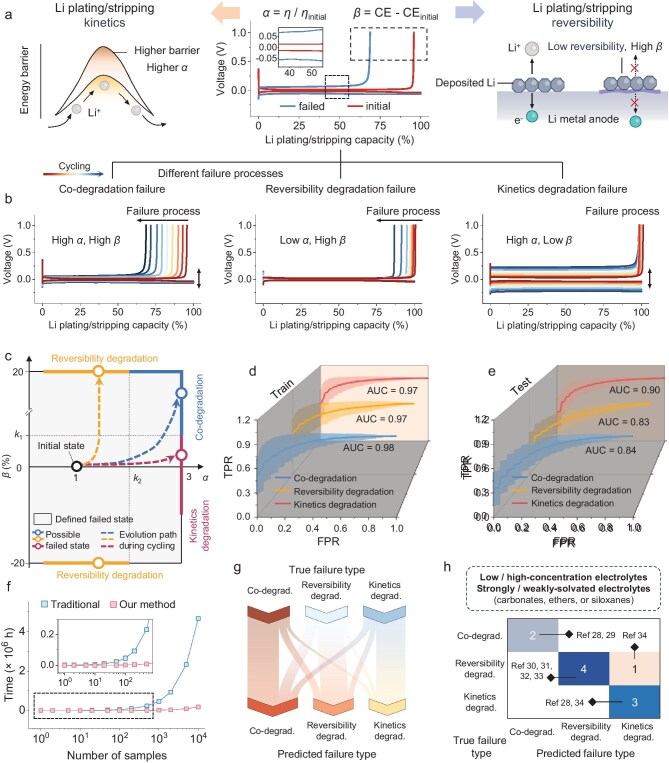
Classification of lithium metal anode failure types and their correlation with initial and evolving curve types. (a) Identification of two dominant factors for lithium metal anode failure. (b) Illustration of the evolution of representative electrochemical curves corresponding to three distinct failure types of the lithium metal anode. (c) Definition of failed states for each type, including specific ranges of *η* and CE changes, *α* and *β*. (d and e) Assessment of AUC values for the ML model, derived from the true positive rate (TPR) and false positive rate (FPR), corresponding to the classification performance of each failure type within the (d) training and (e) testing datasets. (f) Analysis of the time required to identify the failure type as the sample size increases. (g) Evaluation of misclassification instances using the developed ML model. (h) Evaluation of misclassification for validation samples using the developed ML model [28–34].

The kinetics and reversibility during lithium plating/stripping are critical factors to consider in various applications of LMBs, and they are reflected in the electrochemical curves by overpotential (*η*) and Coulombic efficiency (CE) [22] (Fig. 2a). We use changes in *η* and CE obtained from the electrochemical curves in lithium half-cell testing as failed state markers *α* and *β* to represent kinetics and reversibility degradation, respectively. Based on this framework, the failure of lithium metal anodes can be generally categorized into three distinct types: kinetics degradation failure (KDF), reversibility degradation failure (RDF), and co-degradation failure (CDF). Each type has a unique lithium plating/stripping curve evolution pattern, as depicted in Fig. 2b and Note S1. Specifically, we established a universal quantitative standard (Fig. 2c, Note S2, and Fig. S1), and categorized the dataset of lithium plating/stripping curves from many lithium half cells with different electrolytes into the three corresponding failure types for further analysis. Each failure type is associated with specific favorable application scenarios (Figs S2–S4 and Note S3).

To delve into the microscopic origins of these failure types, we conceptualized the failed state of the lithium metal anode as a composite of the initial state, repetitive units, and cumulative quantities (Fig. S5a). With this understanding, knowing any three of these four aspects allows us to deduce the fourth. For example, by knowing the initial state, repetitive units, and the failed state, we can infer the cumulative quantities, which in our study are *α* and *β*. This insight means that the degradation of kinetics and reversibility can be traced from the initial cycles and curve evolution types. We specifically chose the first two cycles for further analysis because they encapsulate the critical early-stage interfacial evolution while minimizing redundancy. The first cycle captures lithium nucleation, early deposition morphology, and dynamic SEI formation, whereas the second cycle reflects lithium deposition/stripping on this evolving SEI framework, during which the SEI undergoes structural adjustments that determine long-term reversibility. By the third cycle, the discharging/charging behavior stabilizes and becomes repetitive, and the SEI enters a steady-state reinforcement phase, contributing limited new mechanistic insights. As a result, from the third cycle onward, the electrochemical curves exhibit a high degree of similarity to the second cycle (Fig. S5b), indicating that the key interfacial evolution has largely been completed by this stage. Using this understanding, we analyzed a large amount of curve data and explored the intricate relationship between the initial cycles and the types of curve evolution. We extracted features that are electrochemically explainable and fully characterize the shape of the curves, encompassing different cycle numbers and various stages, including both absolute and relative values of capacity and voltage (Table S1). Notably, these features are universally applicable and easily extracted, making them highly practical for broader use. These explainable features are crucial as inputs for our model to predict failure types, as they not only capture the essence of the failure process but also provide a clear pathway to decipher the failure mechanisms and trace the early-stage microscopic origin of the observed failure types.

We selected the random forest-based algorithm due to its suitability for structured tabular datasets typical of electrochemical curve data (Note S4). The model yielded a high overall AUC of 0.98 for the training dataset, as depicted in Fig. 2d. This overall score, along with balanced AUC and high prediction accuracy of 0.91, 0.87, and 0.89 for three types (Fig. S6), indicates the reliability of the previous classification criteria and feature engineering. The overall AUC for the testing dataset was 0.86, with well-balanced AUC values of 0.84, 0.83, and 0.90 for the three distinct failure types (Fig. 2e). This outcome reinforces the hypothesis that the initial processes occurring during the initial two cycles are indeed indicative of the intrinsic factors that influence battery failure. This also suggests that the features extracted from the initial two cycles can serve as electrochemical fingerprints for distinguishing failure types. Importantly, this approach not only enhances our comprehension of the underlying mechanisms but also provides a valuable predictive tool for anticipating potential failures in lithium metal anodes. Predicting failure types in advance saves considerable time compared to traditional methods, eliminating the need for a lengthy battery cycling process (Fig. S7 and Table S2). As the number of samples increases, the advantages of using the prediction model become even more pronounced (Fig. 2f).

To further evaluate the predictive model, we analyzed the misclassification results (Fig. 2g). Notably, there is a relatively high incidence of misclassification where RDF and KDF types are incorrectly categorized as CDF-type. This could be attributed to the fact that the CDF encompasses characteristics of both RDF and KDF. Conversely, there is a comparatively lower rate of misclassification between RDF and KDF types, likely due to the distinct nature of their failure types. These results obtained are in full accordance with established classification criteria, thereby validating the model's capabilities. To substantiate the reliability of the selected features and the generalization ability of the predictive model, we conducted a validation exercise. A diverse array of electrolytes (Table S3), previously reported in the literature [28–34], was chosen for their variety in both compositional and solvation structures. They include low-concentration, weakly-solvated, and high-concentration electrolytes based on carbonates, ethers, or siloxanes. Using these electrolytes, we assembled a series of half cells to obtain corresponding electrochemical curves and identified their failure types (Fig. S8). The validation process achieves low misclassification (Fig. 2h), thereby affirming the efficacy of our feature engineering and the robustness of the developed predictive model. As the features and model are universally applicable and user-friendly, this method can serve as a practical tool for a wide range of applications.

### Early-stage microscopic origins for failures

**Figure 3.**
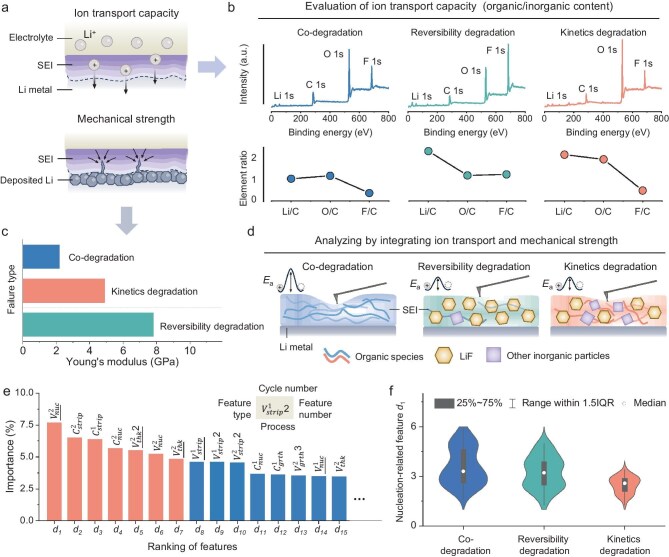
Investigating early-stage microscopic origins for failure of lithium metal anode from SEI characterization and ML model. (a) Key factors influencing the properties of the SEI in lithium metal anodes. (b) Elemental composition analysis of Li, C, and F within the SEI formed on the lithium metal anode for each failure type. (c) Measurement of Young's modulus of the SEI formed on the lithium metal anode for each failure type. (d) Schematic representations of the SEI structures formed on the lithium metal anode, differentiated by the type of failure. (e) Evaluation of the importance of various features for the ML model. Only a selection of the top-ranked features is presented, with underlined features representing relative values and non-underlined features indicating absolute values. (f) Distribution analysis of nucleation feature *d*_1_ for each failure type.

To gain a deep understanding of the failure mechanisms for each type, it is essential to grasp the prediction logic inherent in our model. This involves identifying the key features that significantly influence our model's predictions. The input features of our model, which reflect lithium plating/stripping behaviors, are primarily influenced by the properties of the SEI, especially its ion transport capacity and mechanical characteristics [35–38] (Fig. 3a). Hence, to better comprehend these features, we initiate a preliminary analysis of the SEI properties of lithium metal anodes.

We first used X-ray photoelectron spectroscopy (XPS) to analyze the chemical compositions of SEIs across different failure types (Fig. 3b, Figs S9, S10, and Table S3), which are indicative of their ion transport capabilities. The SEI of CDF-type contains a high proportion of organic components, characterized by a low lithium (Li) content and a high carbon (C) content [39]. This composition suggests a low ionic conductivity. In stark contrast, RDF and KDF types exhibit a higher Li/C ratio, indicative of a higher proportion of inorganic components [39], which are likely to enhance ion conductivity. Notably, the fluorine (F) content, predominantly in the form of lithium fluoride (LiF), was significantly higher in the RDF-type, implying a substantially higher Young's modulus for its SEI. To ensure the representativeness of our selected samples for characterization, we also investigated the chemical compositions of SEIs formed in alternative electrolytes associated with each failure type (Figs S11, S12, and Table S3). The results that are almost consistent with our preliminary findings in terms of element content, as shown in Fig. S13, confirm the reliability of our sample selection.

Mechanical property characterization using atomic force microscopy revealed that the average Young's modulus of RDF-type SEI was indeed exceptionally high, reaching up to 7.8 GPa, followed by KDF-type at 4.9 GPa, with CDF-type being the lowest at 2.2 GPa (Fig. 3c and Fig. S14). We also conducted an additional set of experiments, the results of which were consistent with our initial findings (Fig. S15). These findings are in alignment with the chemical composition trends observed for each SEI type, as summarized in Fig. 3d. The predominance of organic components in CDF-type SEI results in both poor ion transport capacity and mechanical strength, while the inorganic dominance, especially the high LiF content in RDF-type SEI, leads to high mechanical strength and improved ionic conductivity. KDF-type SEI, with a high inorganic content but a moderate amount of LiF, exhibits high ionic conductivity but moderate mechanical strength. Based on the analysis of multiple samples, the fluorine content of SEI in KDF-type likely falls within 5%–8% (Figs S13 and S16).

The distinct SEIs give rise to varied lithium plating/stripping behaviors, reflected in the distinct initial cycle curves. We dissected the features extracted from these curves, categorizing them based on the processes of lithium nucleation, initial growth, later thickening, and stripping during the initial two cycles (Fig. S17). Distributions and further explanations of highly ranked features are shown in Fig. S18 and Note S5. While two highly-ranked stripping features, *d*_2_ and *d*_3_, are widely acknowledged for their significance [40,41], we recognize that the lithium stripping process is directly influenced by lithium plating. Therefore, we have chosen to focus on the characteristics of the lithium plating curve, and this focus aims to uncover the intrinsic and fundamental factors that influence the failure. The ML model's feature importance ranking reveals that highly ranked target features encompass the lithium nucleation and thickening processes (Fig. 3e). This insight underscores the pivotal role that these two stages play in dictating the type of failure.

Among them, highly ranked features reflecting lithium nucleation kinetics *d*_1_, *d*_4_, and *d*_6_ are intricately linked to the SEI properties. In this context, we focus on the highest-ranked feature, *d*_1_. KDF-type shows significantly lower relative nucleation overpotential *d*_1_ than the others (Fig. 3f). Conversely, the low ionic conductivity of CDF-type and the high Young's modulus of RDF-type account for their elevated nucleation overpotentials. A rigid interface generates sustained high local pressure, which elevates the nucleation barrier and, consequently, increases the nucleation overpotential [42]. The trends observed in our study support previous research conclusions that high ionic conductivity and low Young's modulus facilitate nucleation [35,43], thereby reinforcing the validity of our findings. The correlation between SEI properties and the statistical trends of nucleation overpotentials across types underscores the representativeness and interpretability of SEI characteristics. While the nucleation stage is relatively well-researched, the lithium thickening process, characterized by an increasing overpotential associated with features *d*_5_ and *d*_7_, remains less understood. Hence, we pursued further studies to elucidate this stage.

**Figure 4.**
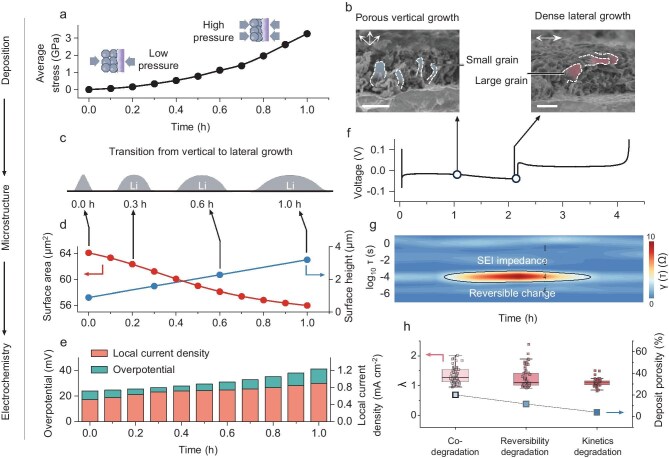
Investigating early-stage microscopic origins for failure of lithium metal anode by detailed analysis of curve features. (a) Simulated average pressure exerted by lithium deposits during the lithium plating process. (b) SEM images depicting the microstructural characteristics of lithium deposits during lithium plating. Scale bars: 5 μm. (c) Simulated evolving microstructure of lithium deposits during the lithium thickening process, transitioning from vertical to lateral growth at the topmost layer. (d) Simulated deposition surface area during the lithium thickening process, transitioning from vertical to lateral growth. (e) Simulation of the local current density (applied current density divided by area) and overpotential change during the lithium thickening process. (f) Discharge/charge curve of the Li||Cu cell during lithium plating/stripping. (g) DRT analysis for *in situ* EIS results during lithium plating/stripping. (h) Distribution of a composite feature λ and porosity for lithium deposits for each failure type.

First, we conducted finite element analysis (FEA) and scanning electron microscopy (SEM) to observe the evolution of the lithium metal anode during the lithium plating process (Fig. 4a and b). Initially, the lithium growth exhibited a relatively porous microstructure, with no significant change in overpotential, and expanded vertically, resulting in a large specific surface area of the lithium particles. However, as plating continued with increasing overpotential, the microstructure at the uppermost layer became increasingly compact, spreading laterally across the plane. Concurrently, the newly formed lithium particles enlarged, with a reduction in their specific surface area [44]. This microstructural change may be influenced by robust ion transport capability, which could benefit from minimal degradation of the SEI during the initial stage, potentially allowing it to exceed the charge transfer rate associated with low current density conditions [45]. Additionally, an increase in the average surface pressure of lithium deposits as the process advances could impede the vertical expansion of lithium, leading to a progressive flattening of the lithium microstructure. This phenomenon is corroborated by several studies that improved deposition morphology through increasing capacities or external pressure [46,47].

Subsequently, we further explored the impact of structural changes during the lithium thickening process, as depicted in Fig. 4c–e. The FEA simulation outcomes indicate that as the microstructure transitions to a flatter configuration, the local current density at the lithium-particle surfaces increases due to the reduction in the specific surface area (Fig. 4d and e). This elevated local current density results in an increased rate of charge transfer, consequently amplifying the concentration gradient within SEI. The resulting concentration polarization augments the SEI impedance, thereby increasing the overall overpotential (Fig. 4e). Therefore, it can be inferred that the rise in overpotential during the lithium thickening process is attributed to the microstructural shift from a porous vertical structure to a dense, flattened one.

To ascertain that the observed increase in overall overpotential stems from resistance within the SEI, we used the distributed relaxation time (DRT) method to disentangle the impedance alterations during the discharging/charging process (Fig 4f and g, and Fig. S19). Our analysis reveals that the SEI impedance is the only parameter that exhibits significant fluctuations with overpotential changes [48], overshadowing the variations in other impedances. It is noteworthy that the SEI impedance changes during discharge and charge processes are reversible. This reversibility of SEI impedance changes can rule out the intrinsic properties of the SEI, such as alterations in ionic conductivity or thickness, as these characteristics are not expected to undergo reversible changes with the lithium plating/stripping process. Consequently, it is postulated that the effective lithium-ion transfer area within the SEI is the only factor that undergoes relatively reversible changes in response to lithium plating and stripping. This finding further substantiates the hypothesis that the increase in overpotential is a direct consequence of the structural evolution, which in turn affects the lithium-ion transfer area.

Additionally, our findings reveal that continuous plating at high capacities leads to a saturation point in the increase of overpotential, which does not escalate further with additional deposition capacity (Fig. S20a). This phenomenon was also observed during our FEA simulation. In the meantime, progressively larger and denser lithium particles with increasing plating capacity were observed (Fig. S20b), suggesting that the saturation value of overpotential may correspond to a sufficiently dense deposition surface and a minimal lithium-ion transfer area for a given system. Given the observed tendency toward dense growth over time, we can infer that at low current densities, where ion transport capabilities significantly exceed charge transfer rates, a more pronounced increase in overpotential may indicate a more porous initial deposition microstructure. Additionally, the earlier the overpotential reaches a saturation point, the sooner the system encounters a pressure threshold, also suggesting a more porous initial microstructure during plating.

Drawing from these insights, we introduce a composite factor, denoted as λ, to fully interpret the curve during the late stage of the lithium plating process. The formula for λ is given in Fig. S21, and a higher value of λ indicates a greater and earlier increase in overpotential during the lithium thickening process, which can be used as an indicator of the deposition microstructure. We conducted a statistical analysis of the λ distribution across three distinct failure types and discovered that the trends in λ values are in alignment with the structural trends observed through SEM in three representative samples (Fig. 4h, Fig. S22, and Note S6), and show a negative correlation with CE (Fig. S23 and Note S7). These findings underscore the expressive capability of λ in reflecting structural changes and highlight the intimate link between structural evolution and the increase of overpotential.

Furthermore, the observed microstructure trends are congruent with the previously determined properties of the SEI and the features reflecting the nucleation process (Fig 3d and f, Fig. S24, and Note S8). KDF-type demonstrates the most favorable microstructure, attributed to its balanced content of inorganic components especially LiF, which supports both good ionic conductivity and mechanical strength. In contrast, RDF-type exhibits a slightly inferior microstructure due to excessive content of LiF, leading to a high Young's modulus but suboptimal ionic conductivity [49]. CDF-type, with the poorest microstructure, has a SEI dominated by organic components that offer poor ionic conductivity and mechanical properties, which are detrimental to both nucleation and deposition microstructure.

**Figure 5.**
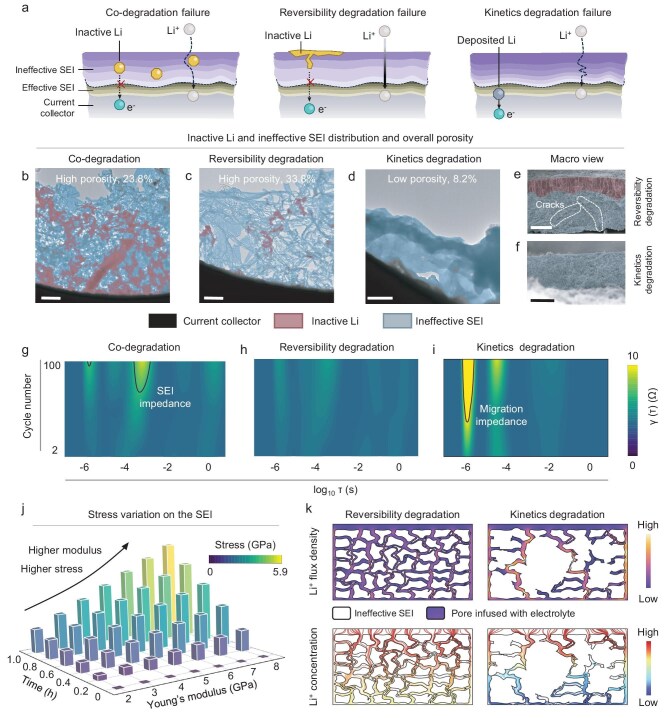
Experimental validation of the progressive impact of early-stage microscopic origins on the failure of lithium metal anodes. (a) Schematic of the failed state characteristics of each identified failure type for lithium metal anodes. (b–d) TEM images for lithium metal anodes post-cycling for each failure type. Scale bars: 2 μm. (e and f) SEM images for lithium metal anodes post-cycling for RDF and KDF types. Scale bars 50 μm. (g–i) DRT analysis for EIS results over cycles. (j) Analysis of stress changes in lithium deposits with varying SEI properties, considering different Young's modulus, during lithium plating. (k) Simulation of lithium-ion flux density and lithium-ion concentration across ineffective SEI with different porosity.

We have now established that the model-identified features with high importance rankings reflect both nucleation and deposit microstructure information, and these are consistent with the properties of the SEI that we previously characterized. To verify the important roles of early-stage nucleation, deposit microstructure, and SEI in influencing failure mechanisms, we further extended the analysis to post-failure (Fig. 5a).

### Progressive impact of early-stage microscopic origins on the failure

To ascertain the relationship between the aforementioned influencing factors and the distinct types of failure, we initially conducted a post-failure structural analysis using transmission electron microscopy (TEM) and SEM, as depicted in Fig. 5b–f, and Figs S25 and S26. The energy-dispersive X-ray spectroscopy mapping data for each failure type corroborate the findings from the preceding XPS analysis, as illustrated in Fig. S27.

Notably, for CDF-type, the generation of substantial inactive lithium during the cycling process is evident in Figs 5b and S26. This discovery directly accounts for the observed gradual decline in the CE curve. The formation of inactive lithium is fundamentally attributed to the suboptimal dendritic microstructure. From a microscopic perspective, the electron paths within the dendrites are confined and elongated, making it challenging to ensure continuous connectivity. This can compromise the reversibility of the plating/stripping process.

To delve into the causes of *η* changes during cycling, we conducted impedance measurements at various stages of the cycling process and subsequently decoupled them using the DRT method (Fig. 5g–i and Fig. S28). The overpotential increase observed in CDF-type is predominantly attributed to the irreversible rise in SEI impedance. This increase stems from the impact of inactive lithium, which creates physical barriers and electrostatic interactions that impede lithium transport within the SEI [50]. Consequently, these factors elongate the overall lithium-ion transport paths, contributing to the observed rise in overpotential.

In contrast, the amount of inactive lithium observed in RDF and KDF types after cycling is relatively low, while the presence of ineffective SEI is pronounced (Fig. 5c–d and Fig. S26). Ineffective SEI originates from the newly formed SEI in each cycle (Fig. S29 and Note S9) [23], therefore, the characteristics of the initial SEI and the microstructure of lithium deposits are pivotal in determining the properties of the ineffective SEI. For CDF-type, the dendritic microstructure is susceptible to SEI damage with each cycling, which, when combined with the relatively low mechanical strength of the SEI, readily leads to the formation of ineffective SEI with high porosity, as illustrated in Fig. 5b. One may question why the ineffective SEI for RDF-type, with a SEI with a high Young's modulus, also exhibits a high porosity (Fig. 5c). It lies in the fact that a high modulus does not equate to a high toughness. It is known that materials with a high modulus often exhibit poor toughness and are prone to fracture. Although RDF-type benefits from an improved microstructure due to its high Young's modulus, a higher Young's modulus corresponds to higher stress under a given deformation (Fig. 5j and Figs S30 and S31), making the SEI more susceptible to damage and resulting in the formation of ineffective SEI with high porosity. Its porous ineffective SEI results in the formation of inactive lithium at the uppermost surface of a thick ineffective SEI layer (Fig. 5e and Fig. S32). This is due to the fact that once a crack extends through the entire layer, lithium preferentially grows on the uppermost surface, avoiding the need to navigate the thick barrier of ineffective SEI. During the subsequent stripping process, the lithium on the surface cannot be reversibly removed due to the narrow and elongated electron paths along the crack, which are prone to disconnection. This phenomenon explains the abrupt change in CE for RDF-type during cycling.

Conversely, KDF-type, with its moderate Young's modulus, is less likely to experience significant stress from lithium volume changes. Coupled with its good lithium microstructure, this results in formation of a dense ineffective SEI (Fig. 5d). Therefore, to enhance the integrity of the SEI, it is advantageous to have a moderate Young's modulus, as excessively high values may counterproductively lead to negative effects. This perspective aligns with previous research, reinforcing the importance of balancing Young's modulus and toughness of the SEI for optimal performance [34]. The dense ineffective SEI in KDF-type prevents the formation of surface inactive lithium (Fig. 5f and Fig. S32), unlike what is observed in RDF-type. However, this dense ineffective SEI may contribute to an increased overpotential. The DRT analysis indicates that the cause of the overpotential increase in KDF-type differs from that in CDF-type, being primarily attributed to an increase in migration impedance [51], as illustrated in Fig. 5i and Fig. S28. We found that the source of migration impedance is the lithium migration within the ineffective SEI/electrolyte composite phase (Figs S33, S34, and Note S10). As illustrated in Fig. 5k, minimal porosity results in a dense and convoluted flow of lithium ions. This condition elongates the ionic transport paths, thereby increasing migration impedance. Consequently, this leads to an elevated overpotential due to increased concentration polarization (Fig. S35). The specific mechanisms for each failure type and their correlation with the electrochemical curves are summarized in Note S11, along with an analysis of the potential causal relationships between each failure type and its cycle life, which is provided in Note S12.

To conclude, the specific influence of the two key factors—lithium nucleation and deposit microstructure—on the failure types is detailed below. These two features collectively reveal the SEI characteristics that are not directly observable from the curve. The SEI, in conjunction with lithium microstructure, dictates the formation of ineffective SEI and inactive lithium. These, in turn, directly influence the charge carrier (lithium-ion and electron) transport paths in the lithium metal anode. Elongated ion paths decrease kinetics, while extended electron paths reduce reversibility, collectively explaining the distinct curve evolution observed in different failure types.

## DISCUSSION

In summary, we identified the failure fingerprints by establishing correlations between the initial lithium plating/stripping behaviors and the eventual failures of a lithium metal anode, leveraging domain knowledge-informed ML to analyze extensive electrochemical curve datasets. Further interpretation of the critical curve features, supported by *in situ* experiments and simulations, revealed that the SEI and lithium deposit microstructure ultimately degrade kinetics and reversibility by disrupting ion and electron transport paths. This work provides new insights into the failure mechanisms of lithium metal anodes through fingerprints hidden in the initial cycles, rather than solely relying on post-mortem analysis.

The findings provide a basis for the development of targeted strategies to mitigate each type of failure. To counteract co-degradation failure, which is characterized by the formation of inactive lithium, strategies such as the recovery of isolated lithium through discharged state calendar aging, as proposed by [52], should be considered. For reversibility degradation failure, which is often associated with a high Young's modulus of the SEI, the incorporation of additives that enhance SEI flexibility is recommended. For instance, the addition of a ‘plasticizer’ in the SEI to improve its mechanical flexibility and toughness [53], thereby reducing the risk of SEI fracture and associated capacity loss. Addressing kinetics degradation failure may involve the use of solvents with a high donor number to dissolve dense ineffective SEI layers [54], creating a more porous structure that facilitates ion transport. While these strategies can delay the onset of failure, it is important to acknowledge that avoiding both kinetic and reversibility issues simultaneously is challenging. Achieving a balance requires an optimal Young's modulus for the SEI, a concept supported by the research of [34].

Another key significance of this work is that the ML model provides a predictive framework for evaluating battery performance and degradation paths, allowing for anticipating potential issues proactively. This predictive ability enables informed decisions about the battery's suitability for varied applications, eliminating the need for lengthy testing and extensive post-mortem characterizations. The model's streamlined design and the ease of feature extraction make it user-friendly for researchers and engineers, improving the efficiency of the research and development process. In this study, we selected the random forest algorithm primarily because it effectively accommodates structured, tabular datasets, such as the electrochemical fingerprint features derived from initial battery cycling curves. Random forest models provide stable predictive performance in the context of moderate-sized datasets while offering transparent feature importance, enabling straightforward mechanistic interpretation.

The data-driven approach presented in this work has demonstrated clear advantages over traditional methodologies. By using extensive datasets and ML, we achieve high predictive accuracy, reducing the time and resource investment compared to traditional failure characterization methods. The ability to derive predictive insights from initial cycling data greatly enhances efficiency, facilitates rapid electrolyte screening, and accelerates practical battery development. However, the data-driven paradigm is not without limitations. Its effectiveness strongly relies on the quality, representativeness, and comprehensiveness of the datasets available. In lithium metal battery research, the availability of large-scale, diversified data remains limited, restricting the generalizability and accuracy of predictions across systems or significantly different operating conditions. Furthermore, purely data-driven models may yield correlations without elucidating underlying causative mechanisms unless rigorously integrated with domain-specific experimental validations, as conducted in our study.

Several promising directions could extend our current framework. First, continuous collection and integration of larger and diversified electrochemical datasets could further enhance accuracy and generalizability. Additionally, leveraging physics-informed synthetic data generation methods based on mechanistic electrochemical principles could supplement limited experimental data, thus providing richer and more diversified training datasets without consuming extra testing resources. Besides, developing specialized large-scale models, such as domain-specific large language models tailored explicitly for lithium metal battery research, may offer significant advantages. It is also promising in constructing a dynamic knowledge graph of failure mechanisms, encompassing SEI properties, microstructural evolution, and electrochemical behaviors, which allows for continuous updates as new data and insights emerge. Furthermore, we believe that further research into the relationship between electrolyte composition and failure mechanisms presents a promising direction for future studies.

## Supplementary Material

nwaf158_Supplemental_File
