# Supplementary material for: Deciphering failure paths in lithium metal anodes by electrochemical curve fingerprints
Source: Natl Sci Rev. 2025 Apr 24;12(7):nwaf158. doi: 10.1093/nsr/nwaf158 (PMC12153722; doi:10.1093/nsr/nwaf158)
Supplement: nwaf158_Supplemental_File [file nwaf158_supplemental_file.pdf]

Supplementary Information

# Deciphering failure paths in lithium metal anodes by electrochemical curve fingerprints


Zhihong Piao,[1,#] Zhiyuan Han,[1,#] Shengyu Tao,[1,#] Mengtian Zhang,[1] Gongxun Lu,[1] Lin Su,[1] Xinru Wu,[1] Yanze Song,[1] Xiao Xiao,[1] Xuan Zhang,[1] Guangmin Zhou,[1]* and Hui-Ming Cheng[2,3,4,]*

[1]Tsinghua Shenzhen International Graduate School, Tsinghua University, Shenzhen 518055, China;

[2]Institute of Technology for Carbon Neutrality, Shenzhen Institute of Advanced Technology, Chinese Academy of Sciences, Shenzhen 518055, China;

[3]Faculty of Materials Science and Energy Engineering, Shenzhen University of Advanced Technology, Shenzhen 518055, China;

[4]Shenyang National Laboratory for Materials Science, Institute of Metal Research, Chinese Academy of Sciences, Shenyang 110016, China

***Corresponding authors.** E-mails: guangminzhou@sz.tsinghua.edu.cn; cheng@imr.ac.cn

[#]Equally contributed to this work.

# Inventory of Supplementary Information

Methods
Supplementary Notes 1-12
Supplementary Figures 1-35
Supplementary Tables 1-3
Supplementary References 1-13

# Methods

## Materials

*Chemicals*

The following chemicals were utilized in this study. Lithium bis(fluorosulfonyl)imide (LiFSI), lithium bis((trifluoromethyl)sulfonyl)azanide (LiTFSI), lithium hexafluorophosphate ($LiPF_6$), and lithium difluoro(oxalato)borate (LiDFOB) were procured from DoDo Chem and used as received. Sorbide nitrate (SN) was purchased from Shanghai Yuanye Biotechnology Co., Ltd. and used as received. Additionally, solvents such as ethylene carbonate (EC), ethyl methyl carbonate (EMC), dimethyl carbonate (DMC), fluoroethylene carbonate (FEC), and methyl 2,2,2-trifluoroethyl ester (FEMC), 1,2-dimethoxyethane (DME) were sourced from DoDo Chem, while 1,1,2,2-tetrafluoroethyl-2,2,3,3-tetrafluoropropylether (TTE) was provided by Macklin. Diethyl carbonate (DEC), diethoxydimethylsilane (DMES), dimethyldimethoxysilane (DMMS), and dimethoxy(methyl)(3,3,3-trifluoropropyl)silane (DMOTFS) were purchased from Aladdin. Prior to their use, all these chemicals were purified using 300 ℃ pretreated molecular sieves, which were also supplied by Aladdin.

*Electrolytes for major characterizations and validation*

Three distinct electrolyte formulations were prepared to investigate the failure types of lithium metal anodes. These include 1 M $LiPF_6$ in EC:EMC (1:1 v/v) + 2% FEC (DoDo Chem), 1 M $LiPF_6$+0.1 M LiDFOB in FEC:FEMC (1:3 v/v), and 1.5 M LiFSI in DMOTFS. To further validate the machine learning (ML) model and to rigorously assess the representativeness of the sample selection and characterization results, the following electrolytes were chosen: 1 M $LiPF_6$ in EC:EMC:DMC (1:1:1 v/v/v), 1 M LiTFSI in EC:DEC (1:1 v/v), LiFSI: DME:TTE, 1:1.2:3 (m/m/m), 1 M $LiPF_6$ in DMC:FEC (8:2 v/v) + 1% SN, 2.1 M LiFSI in DMES, 1 M $LiPF_6$ in EC:DMC (1:1 v/v), 1 M LiTFSI in DME:DOL (1:1 v/v) + 5% $LiNO_3$, LiFSI: DME: TTE, 1:9:27 (m/m/m), 1 M $LiPF_6$ in DMC:FEC (8:2 w/w), 1.5 M LiFSI in DMMS. The specific electrolyte formulations to obtain the electrochemical curve data utilized in the development of the ML model are not disclosed in this work. However, we can confidently assert that the electrolytes encompass a wide array of compositions and characteristics to ensure a robust and diverse dataset for our ML analysis.

## Cell assembly and electrochemical tests

*Electrochemical Cell Assembly*

The 2032-type Li||copper (Cu) coin cells were fabricated utilizing cases sourced from Guangdong Canrd New Energy Technology Co. Ltd. A 450 μm lithium foil served as the reference electrode, 19 μm copper as the working electrode, and Celgard 2500 as the separator, all supplied by Guangdong Canrd New Energy Technology Co., Ltd. Each electrochemical test utilized 50 μL of electrolyte. The assembly of all cells was meticulously conducted within an argon-filled glovebox to ensure an inert atmosphere.

*Electrochemical Testing and Data Collection*

For the initial data collection necessary for ML model development, 162 Li||Cu cells were cycled using multipathway battery testers from LAND (Wuhan, China) or Neware (Shenzhen, China) at ambient temperatures. The cycling was performed at current density and capacity not exceeding 1 mA $cm^{-2}$ and 1 mAh $cm^{-2}$, respectively. EIS measurements were conducted on a Biologic SP150. The Li||Cu cells were subjected to EIS tests with an AC amplitude of 10 mV, covering a frequency range from 100 mHz to 1 MHz. In situ EIS data were acquired by performing EIS measurements at specific intervals during the cycling process, conducted at a current density of 0.5 mA $cm^{-2}$. The DRT analysis was conducted according to a reported method [1].

**Characterizations**

The chemical composition of SEI was analyzed by X-ray Photoelectron Spectroscope (PHI 5000 VersaProbe II, Escalab Xi+). Young's modulus of SEI was tested on an Atomic Force Microscope (Bruker Dimension Icon) based on the Derjaguin-Muller-Toporo model. Morphology of lithium deposit and ineffective SEI was observed using a Scanning Electron Microscope (Hitachi SU8010) and Transmission Electron Microscope (Talos F200X G2, 200 kV) operated at 200 kV. For porosity quantification, observed images were imported into ImageJ software for further analysis. The samples for TEM were cycled for 10 cycles due to the specific sample preparation requirements. The porosity was calculated using the following formula: Porosity = $A_{Pore} / A_{total}$ ×100%, where $A_{Pore}$ is the total area of the pores, and $A_{total}$ is the total area of the analyzed region. All batteries were carefully disassembled within an argon-filled glovebox to maintain a controlled environment and prevent contamination. The electrodes were thoroughly washed with DME three times to ensure the removal of any residual electrolytes and impurities, thereby facilitating a more accurate analysis.

**ML**

*The random forest classification*

The Random Forest classifier is an ensemble learning method that operates by constructing multiple decision trees during the training process. Each tree is built using a random subset of the training data through a process known as bootstrap aggregation or bagging. The final prediction is made based on the majority vote across all trees for classification tasks.

Given a dataset $D$ with features $\mathbf{X} = \{\mathbf{x_1}, \cdots, \mathbf{x_{n-1}}, \mathbf{x_n}\}$ and labels $\mathbf{y} = \{y_1, \cdots, y_{n-1}, y_n\}$, the RF model generates $T$ decision trees. For each tree $t$, a bootstrap sample of the training data is selected, and the model is trained on that subset. The prediction for a sample $x$ is given by the majority vote of the trees:

$$\hat{y} = \mathrm{mode}(y_t(x)), t = 1,2, \dots, T$$

where, $T = 50$ is the number of trees, $y_t(x)$ is the prediction of tree $t$ for sample x. $\hat{y}$ is the final predicted label based on the majority vote. The entropy criterion is used to measure the quality of splits in each decision tree. At each split, a random subset of features is considered, controlling overfitting. The RF classifier aggregates predictions across all trees to improve accuracy and reduce the variance of individual decision trees.

*The random forest classification*

Monte Carlo (MC) simulations are used to evaluate the performance and robustness of the RF model. Each simulation run involves resampling the data, training the RF classifier on different bootstrap samples, and evaluating its performance. For each of the 200 MC runs, the following procedure is followed: (1) A random seed is set to ensure that each run uses a different random sample of the dataset. (2) The dataset is split into training and testing sets using a stratified shuffle split to maintain the distribution of labels. (3) The RF model is trained on the training set and tested on the testing set, with predictions recorded. The MC experiments are configured with the following parameters. The number of Monte Carlo runs is set to 200. The proportion of the dataset used for testing and training is 20% and 80%, respectively. This simulation allows us to assess the stability and performance of the model over multiple random trials.

*The feature importance*

In a Random Forest model, feature importance is a measure of how valuable each feature is in making accurate predictions. It is calculated as the decrease in node impurity (entropy in this case) across all trees when the feature is used in a split. The importance of a feature $x_j$ is computed as:

$$\mathrm{I}(\mathrm{x_j}) = \frac{1}{\mathrm{T}}\sum_{\mathrm{t}=1}^{\mathrm{T}} \Delta \mathrm{I_t}(\mathrm{x_j})$$

where, $\mathrm{I}(\mathrm{x_j})$ is the importance of feature $\mathrm{x_j}$. $\mathrm{T} = 50$ is the number of trees, $\Delta\mathrm{I_t}(\mathrm{x_j})$ is the decrease in impurity when splitting on feature $\mathrm{x_j}$ in tree $\mathrm{t}$. The final importance score for each feature is averaged across all Monte Carlo runs to obtain a more reliable measure of its contribution to the model.

*Evaluation metrics*

We use the ROC curve to evaluate the model prediction results. The ROC curve is generated by plotting the true positive rate (TPR) against the false positive rate (FPR) at different classification thresholds. These metrics are averaged across all Monte Carlo runs to give a comprehensive evaluation of the model's performance. The TPR and FPR are given by:

$$\mathrm{TPR} = \frac{\mathrm{TP}}{\mathrm{TP} + \mathrm{FN}}, \mathrm{FPR} = \frac{\mathrm{FP}}{\mathrm{FP} + \mathrm{TN}}$$

where, True Positive (TP) refers to instances where the model correctly predicts a sample as belonging to its actual failure mode. For example, if a battery truly experiences a certain failure mode and the model correctly classifies it as such, this counts as a TP. Conversely, False Positive (FP) occurs when the model incorrectly predicts a sample as belonging to a failure mode when it actually does not. For instance, if the battery is not experiencing the failure mode but the model predicts that it is, this counts as an FP. Similarly, False Negative (FN) refers to instances where the model fails to recognize that a sample belongs to a specific failure mode. In this case, a battery that is truly experiencing the failure mode is incorrectly classified as not belonging to that mode, resulting in an FN. Finally, True Negative (TN) refers to instances where the model correctly predicts that a sample does not belong to a particular failure mode, meaning the battery does not experience the failure mode and the model accurately reflects this.

**FEA simulations**

Using COMSOL Multiphysics 6.2, we employed finite element analysis to simulate the deposition behavior of lithium anodes and the evolution of stress. The following are the control equations used in the simulation:

*Mass transport*

In the electrolyte, ion transport is driven by both migration in response to the electric

field and diffusion due to concentration gradients, as described by the Nernst-Planck equation:

$$N_i = -D_{e,i}\left(\nabla C_{e,i} - \frac{z_i F C_{e,i}}{RT}\nabla \phi_e\right)$$

where $N_i$ is flux, $D_{e,i}$, $z_i$ and $C_{e,i}$ are the diffusion coefficient in the electrolyte, charge, and concentration of species $i$, respectively. $F$ is Faraday's constant, $R$ is the ideal gas constant, $T$ is the Kelvin temperature and $\phi_e$ is the electrolyte potential.

The ions present within the electrolyte adhere to the principles of mass conservation and charge conservation, which can be expressed as:

$$\frac{\partial C_{e,i}}{\partial t} + \nabla \times N_i = 0$$

$$\sum_i z_i\, C_{e,i} = 0$$

where $z_i$ is the valence of each species in the electrolyte.

*Charge transfer*

Fundamentally, lithium ions are transported from the bulk solution to the anode surface, where they are reduced to lithium atoms. At the electrolyte-anode interface, the deposition process of lithium ions can be described by the simplified reaction:$Li^+ + e^- \rightleftharpoons Li$

The electrochemical reaction of charge transfer occurring at the electrolyte-anode interface can be quantified by local current density, which is given by the Butler-Volmer equation:

$$i_{local} = i_{ex,j}[\prod\left(\frac{C_{e,i}}{C_{i,ref}}\right)^{pi,j} \exp\left(\frac{\alpha_{a,j}F\eta_j}{RT}\right) - \prod\left(\frac{C_{e,i}}{C_{i,ref}}\right)^{qi,j} \exp\left(\frac{\alpha_{c,j}F\eta_j}{RT}\right)$$

$pi,j = si,j$ for oxidized species, $pi,j = -si,j$ for reduced species.

where $i_{ex,j}$ is exchange current density, $\alpha_{a,j}$ and $\alpha_{c,j}$ are the anodic and cathodic charge transfer coefficients, respectively, $C_{i,ref}$ is the reference concentration and $C_{e,i}$ is the concentration of each species i near the interface between electrode and electrolyte, $\eta_j$ is overpotential, which can be calculated from:

$$\eta = \phi_s - \phi_e - U_{eq}$$

where $\phi_s$ is the solid phase potential, $U_{eq}$ is the equilibrium potential of the reaction.

*Lithium deposition Morphology*

The local deposition rate is directly related to the local current density, therefore, the

boundary condition at the interface is given by:

$$N_{Li^+} \cdot \boldsymbol{n} = -\frac{i_{local}}{F}$$

where $\boldsymbol{n}$ is the normal vector of the boundary.

The resulting deposition morphology was assumed to occur in the normal direction to the boundary with a velocity $v_n$:

$$v_n = N_{Li^+} \frac{M}{\rho}$$

where $M$ and $\rho$ is the molar mass and density of lithium metal.

To simulate the morphology of lithium deposits, we set the interface between the anode and electrolyte as a free boundary. The deposition thickness of lithium was used as the displacement in the normal direction and can be expressed by:

$$v_n = \boldsymbol{n} \frac{dz}{dt}$$

*Mechanical simulation*

For mechanical performance simulation, the displacement of the electrode surface is calculated according to the above equations, and the kinematic stress-strain constitutive relation is given by Hooke's Law:

$$\sigma_{ij} = \frac{E}{1+\nu} \nabla \vec{\ell} + \frac{2\nu E}{1-2\nu}$$

where E is Young's modulus, $\vec{\ell}$ is the displacement of the interface and ν is Poisson's ratio.

## Supplementary Notes

**Supplementary Note 1. Description of curve evolutions of each failure type.**

In the CDF-type curve evolution, as the cycle number increases, $\eta$ increases and CE decreases, indicating a high $\alpha$ and $\beta$ with simultaneous degradation in both kinetics and reversibility. RDF-type shows no significant increase in $\eta$ over cycling, but experiences a sudden drop in CE, suggesting good kinetic maintenance (low $\alpha$) but poor reversibility (high $\beta$) for extended cycling. The KDF-type exhibits a gradual increase in $\eta$ (high $\alpha$) during cycling while CE remains almost constant (low $\beta$), suggesting a commendable level of reversibility coupled with suboptimal kinetic performance. The evolution of electrochemical curves indicates that, under low current conditions, kinetics degradation and reversibility degradation are independent of each other.

**Supplementary Note 2. A universal quantitative standard for categorizing failure types.**

The failure was defined as a CE change of over 20% or an $\eta$ increase to three times its initial value, based on the distribution of CE and $\eta$ changes up to the point of failure, with outliers not clustered around the main peak regarded as failed states (Figure S1). The comprehensive range of these failure types is graphically depicted, with $k_1$ and $k_2$ defined as 2. Ideally, when $\alpha = 1$ and $\beta = 0$, it indicates that there are no changes in kinetics and reversibility at the point of failure. This ideal scenario serves as a baseline, with the clustered data points considered within the error margin of fluctuation. For simplicity, integer values were chosen as critical thresholds, leading to the final determination of $k_1$ and $k_2$ as 2. Even if we acknowledge that the defined quantitative criteria may have some margin of error, it is acceptable because there are only a few samples differing among various reasonable standards, which do not significantly impact the overall model.

**Supplementary Note 3. Evaluation of failure types to assess their suitability for different application scenarios.**

A statistical analysis of cycle life revealed that the CDF-type, with a significantly lower cycle life compared to the other types (Figure S2), is not ideal for long-term cycling. For a deeper evaluation of RDF and KDF types, we selected representative electrolytes for each and tested the tolerance of lithium metal half-cells for current and capacity. As current densities increased, RDF-type showed a notably lower $\eta$ compared to KDF-type (Figure S3), indicating its superior kinetic tolerance. KDF-type exhibited a higher and narrower distribution of CE with increasing

capacity and denser lithium plating indicating improved reversibility (Figure S4). These findings align with the curve evolution tendencies of these types. In summary, RDF-type is better suited for fast-charging scenarios, while KDF-type is more appropriate for high-capacity applications.

**Supplementary Note 4. Rationale for selecting the random forest model.**

Unlike advanced deep neural networks or large language models, random forest balances interpretability and predictive accuracy without necessitating excessively large datasets. Other models, such as deep learning methods, typically require extensive training data, which is scarce in the lithium metal battery domain. Given the relatively limited dataset size available, employing complex models would likely lead to overfitting, compromising predictive robustness.

**Supplementary Note 5. Physical and chemical mechanisms underlying the key electrochemical features.**

These highly ranked features are strongly correlated with the fundamental phenomena of the lithium metal anode: a) Nucleation overpotential $d_1$ and $d_6$: A higher nucleation overpotential indicates a larger nucleation energy barrier, which may arise from a higher Young's modulus or lower ionic conductivity of the SEI, making it more difficult for lithium-ions to undergo reduction deposition at the electrode interface. b) Nucleation capacity $d_4$: A larger nucleation capacity suggests a longer transition phase from nucleation to bulk deposition, reflecting poorer early-stage nucleation quality or more harsh growth conditions, which hinder a rapid transition to stable growth. c) Stripping capacity $d_2$ and $d_3$: This parameter directly reflects the reversibility of the lithium plating/stripping process. A higher stripping capacity indicates a significant suppression of inactive lithium formation and reflects a more dense and uniform deposition morphology. Notably, in the second cycle, as the SEI structure relatively stabilizes, the measured stripping capacity more accurately reflects the intrinsic reversibility of the deposition. d) Later-stage deposition overpotential $d_5$ and $d_7$: As discussed later in the main text, these parameters are strongly correlated with the morphology of the lithium deposit. These features contribute significantly to predicting battery failure because they directly reflect three core failure-inducing factors: SEI characteristics, nucleation kinetics, and deposition morphology evolution. These microscopic factors ultimately lead to macroscopic failure.

**Supplementary Note 6. Lithium deposit structural trends of three representative samples for each failure type.**

In the case of CDF-type, although the lithium plating appears relatively homogenous in optical photographs, SEM reveals a distinctly dendritic and porous microstructure. For RDF-type, there is an improvement in localized microstructure; however, the lithium particles remain small, and optical photographs also indicate inhomogeneities in the plating. Conversely, the KDF-type exhibits larger lithium particles with reduced porosity in SEM images, and the optical photographs corroborate this with a homogeneous and bright lithium surface.

**Supplementary Note 7. Negative correlation between the trend of λ and CE across the three failure types.**

This inverse relationship can be attributed to several factors associated with the microstructure of lithium plating. A loose microstructure characterized by high porosity and a high λ value implies a larger specific surface area, which can lead to greater lithium loss during the formation of SEI [2]. Additionally, this microstructure is more susceptible to the generation of inactive lithium, which further impacts the CE [2].

**Supplementary Note 8. Further analysis of the distribution of microstructure and nucleation features.**

Further analysis, based on the distribution of microstructure and nucleation features (Figure S24), reveals a direct correlation for CDF-type: poorer nucleation leads to a worse microstructure. This suggests that a large nucleation overpotential for this type is primarily due to the low ionic conductivity of the organic components, which are not conducive to both effective nucleation and favorable microstructure. Conversely, RDF-type is likely to have improved microstructure when coupled with poorer nucleation, which aligns with its high content of inorganic components, particularly LiF. This composition is advantageous for achieving a dense microstructure but is less favorable for nucleation. By integrating the analysis of microstructure with the nucleation features, we have further substantiated that the nucleation overpotentials for CDF and RDF types are influenced by distinct aspects of SEI properties. CDF-type's overpotential is primarily affected by the poor ionic conductivity of its organic components, while RDF-type's overpotential is influenced by the presence of inorganic components like LiF that promote dense microstructure but hinder nucleation.

**Supplementary Note 9. Formation of ineffective SEI.**

The formation of ineffective SEI is attributed to the loose contact between the SEI and the current collector following the stripping of a significant volume of lithium

(Figure S29). The volumetric changes in lithium are substantially greater than the reversible deformation capacity of the SEI, leading to the detachment of some SEI components. This detachment renders them incapable of functioning effectively as an interphase for the subsequent lithium plating process. That is, the ineffective SEI originates from the newly formed SEI in each cycle once it loses contact with the newly plated lithium particles.

**Supplementary Note 10. Origin of variation in migration impedance across different failure types.**

Lithium-ion migration involves the movement of lithium-ions across the electrolyte and the composite phase of the ineffective SEI/electrolyte, facilitated by the presence of pores in the ineffective SEI that allow electrolyte infiltration [3]. To determine if the increased migration impedance in KDF-type is due to the consumption of charge carriers in the electrolyte each cycle, we analyzed the electrolyte composition post-cycling for KDF-type. We made a hypothetical calculation under extreme conditions, assuming that lithium loss in each cycle is solely due to the decomposition of lithium salts to form a new SEI. Based on the CE of each cycle, we estimated that the lithium salt concentration in the battery after 160 cycles would decrease from 1.5 M to 1.2 M. We then assembled a cell using a degraded electrolyte with a salt concentration of 1.2 M, cycled it, and measured EIS. Following DRT decoupling, we found that both the migration impedance and overall overpotential were significantly lower than the actual values obtained after 100 cycles (Figures S33 and S34). This finding led us to rule out the impact of changes in the liquid-phase components on migration impedance. Therefore, another potential source of migration impedance could be the lithium migration within the ineffective SEI/electrolyte composite phase.

**Supplementary Note 11. Summary of specific mechanisms for each failure type and their correlation with the electrochemical curves.**

In the CDF-type, the low ionic conductivity and poor mechanical strength of the SEI lead to dendritic growth, which subsequently results in the formation of inactive lithium and a decline in CE. This also elongates the lithium-ion transport path, deteriorating kinetics, as evidenced by the increased SEI impedance in the DRT analysis. The RDF-type is characterized by an ineffective SEI with high porosity, formed due to the brittle nature of the SEI that is prone to cracking. This causes the accumulation of significant amounts of inactive lithium on the surface of the ineffective SEI, leading to poor reversibility. However, the high porosity of the ineffective SEI does not severely impede lithium-ion transport, meaning that kinetics

and overpotential remain stable during cycling. The KDF-type exhibits the most balanced SEI characteristics, with high ionic conductivity and a moderate Young's modulus. This minimizes inactive lithium formation and maintains good reversibility. However, as the ineffective SEI becomes relatively dense over time, it negatively impacts lithium-ion transport, resulting in increased migration impedance (as shown in the DRT analysis), and consequently, higher overpotential during long-term cycling.

**Supplementary Note 12. Causal relationships between each failure type and its cycle life.**

As shown in Figure S2, the CDF-type shows a relatively shorter cycling life because the failure is primarily due to the gradual accumulation of inactive lithium from lithium dendrite growth. This process occurs during every cycle, and the combined degradation of both the kinetics and reversibility in each cycle leads to a shorter lifespan. The RDF and KDF types exhibit longer cycle life. For RDF-type, degradation only occurs after long-term cycles as the morphology gradually worsens, leading to dendrites penetrating the ineffective SEI layer and forming inactive lithium on the surface. For KDF-type, the impact on the lithium-ion transport path is minimal when the ineffective SEI is thin. As the SEI thickens over time, the overpotential increases rapidly, accelerating failure. Therefore, the failure process in RDF and KDF types is relatively slower, which contributes to their longer cycle life.

## Supplementary Figures

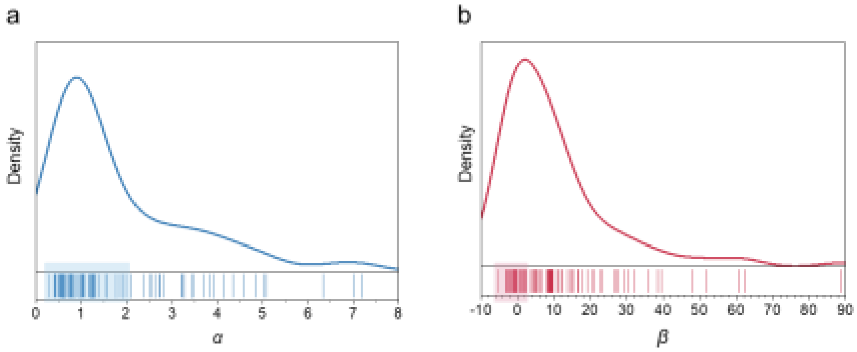


Supplementary Figure 1. (**a**) $\alpha$ distribution with $\beta$ fixed at 20% for various cells. (**b**) $\beta$ distribution with $\alpha$ fixed at 3 for various cells.

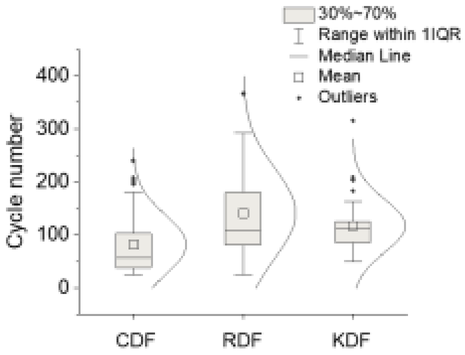


Supplementary Figure 2. Presentation of the cycle life distribution for each failure type.

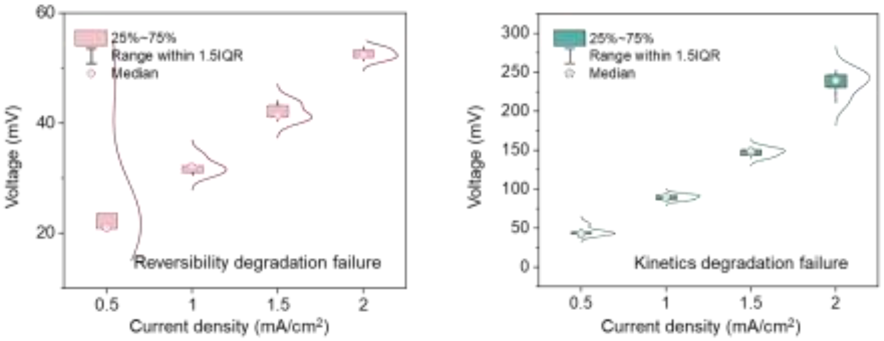


Supplementary Figure 3. Variation in $\eta$ for Li||Cu cells characterized by RDF and KDF types under varied current densities.

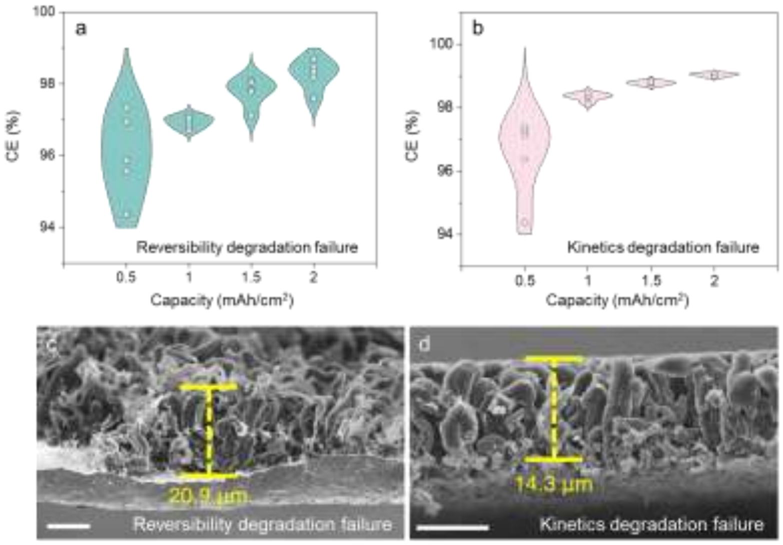


Supplementary Figure 4. (**a and b**) CE profiles for Li||Cu cells characterized by RDF and KDF types with varied deposition capacities. (**c and d**) Cross-sectional SEM images of lithium deposits at 2.5 mAh cm$^{-2}$. Scale bars 10 μm.

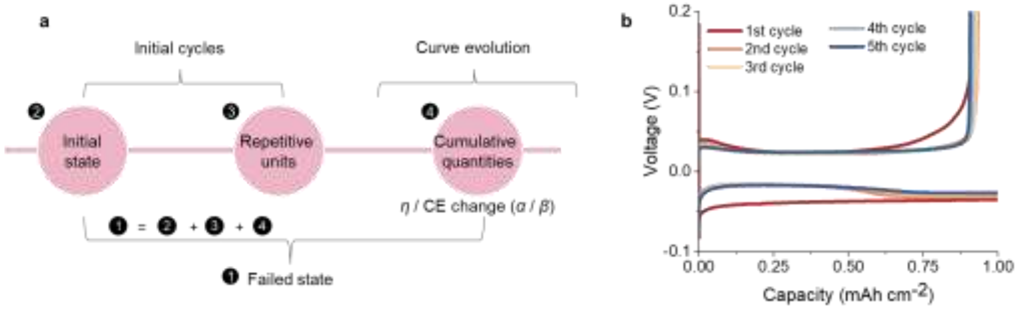


Supplementary Figure 5. (**a**) Schematic of logic for determining ML model inputs for anode failure prediction. (**b**) Representation of the first five cycles.

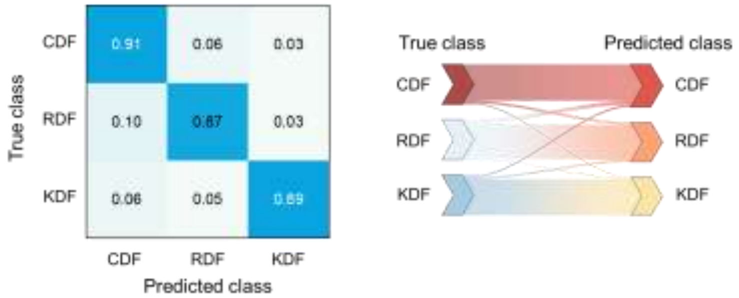


Supplementary Figure 6. Confusion matrix and Sankey diagram for the evaluation of misclassification among failure types within the training dataset.

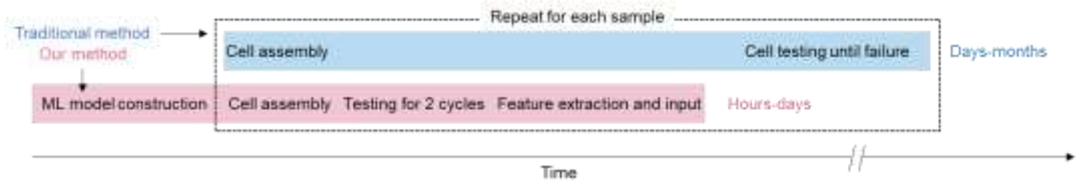


Supplementary Figure 7. Efficiency analysis and comparison of time required to classify failure types utilizing traditional and our method.

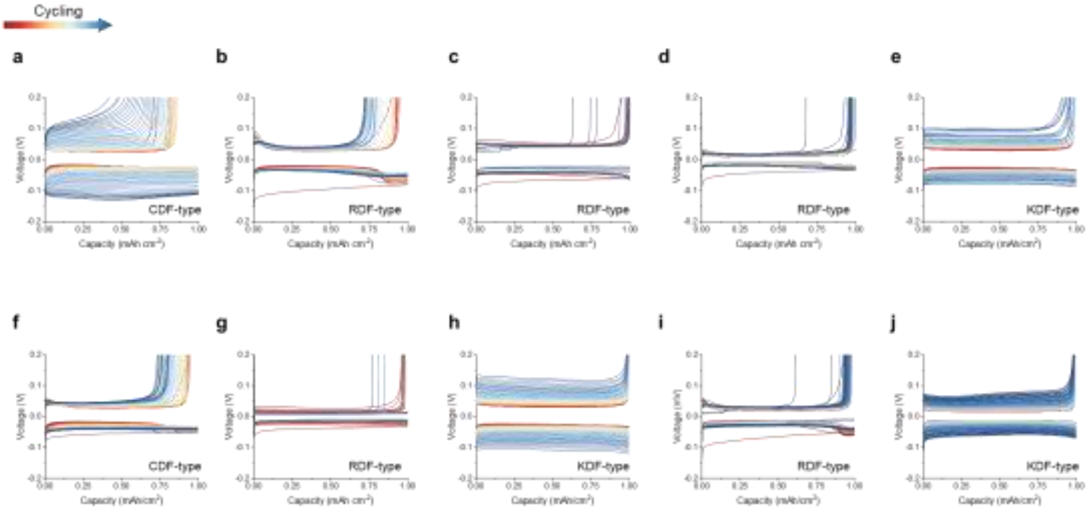


Supplementary Figure 8. Curve evolutions of Li||Cu cells for validation samples. Chosen electrolytes are listed in Supplementary Table 3.

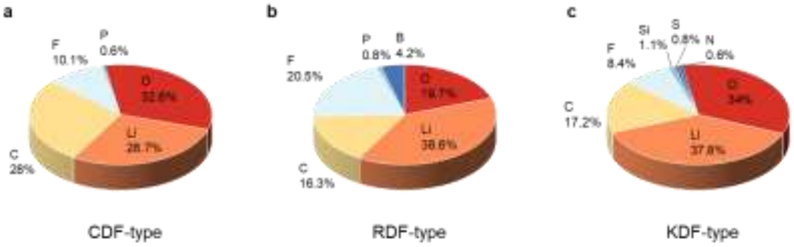


Supplementary Figure 9. Elemental composition of the SEIs formed on lithium metal anodes for each failure type.

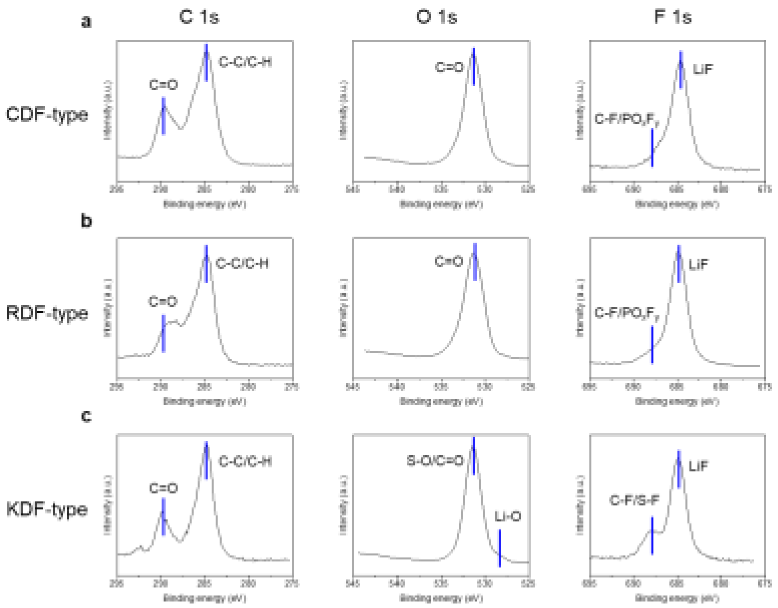


Supplementary Figure 10. XPS spectra of C 1s, O 1s, and F 1s for the SEIs in lithium metal anodes for each failure type.

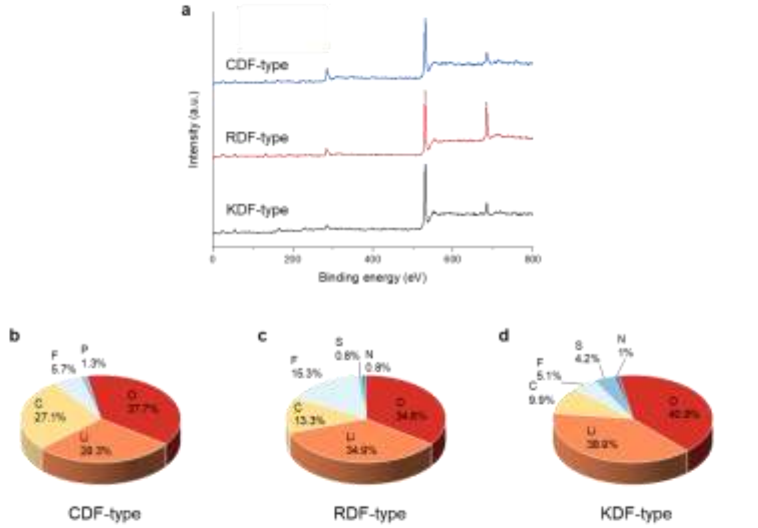


Supplementary Figure 11. XPS survey spectra and detailed elemental composition of the SEIs in validation samples for each failure type.

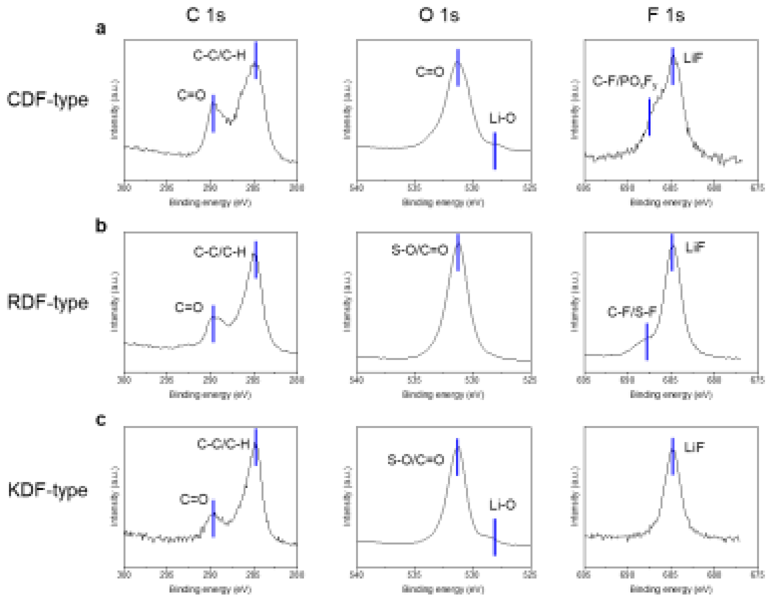


Supplementary Figure 12. XPS spectra of C 1s, O 1s, and F 1s for the SEIs in validation samples for each failure type.

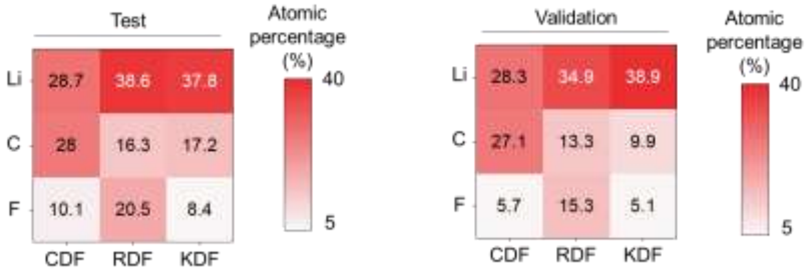


Supplementary Figure 13. Elemental composition comparison of Li, C, and F in the SEIs in test and validation samples for different failure types.

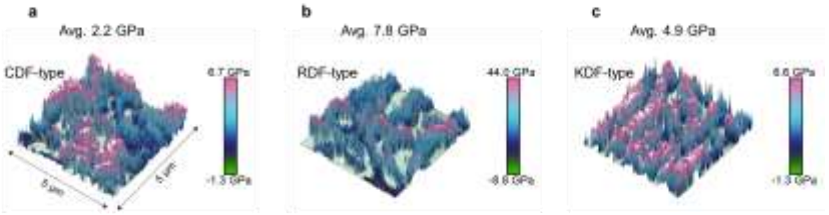


Supplementary Figure 14. Young’s modulus distribution mapping across the SEIs on lithium metal anodes for each failure type.

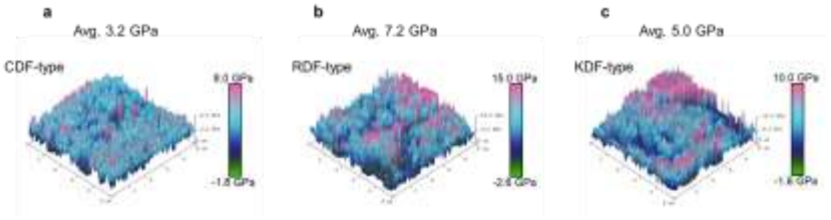

Supplementary Figure 15. Young's modulus distribution mapping across the SEIs in validation samples for each failure type.

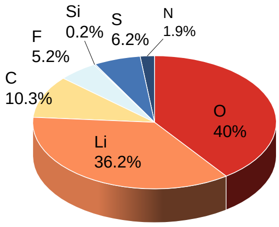


Supplementary Figure 16. Elemental composition of the SEI on the lithium metal anode in 1.5M LiFSI in DMMS (additional sample used for elemental composition analysis).

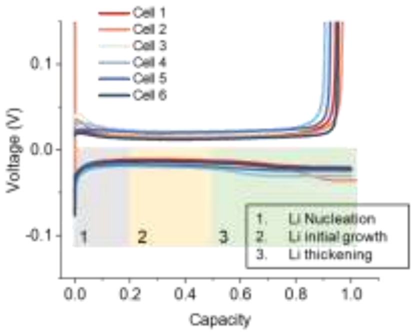


Supplementary Figure 17. Voltage curve characteristics in the second cycle for randomly selected cells with various electrolytes, and classification of lithium plating processes.

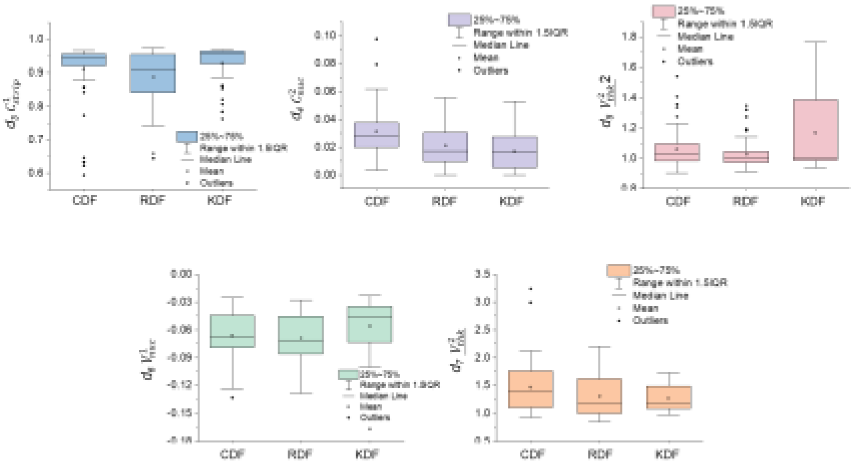


Supplementary Figure 18. Distributions of highly ranked features.

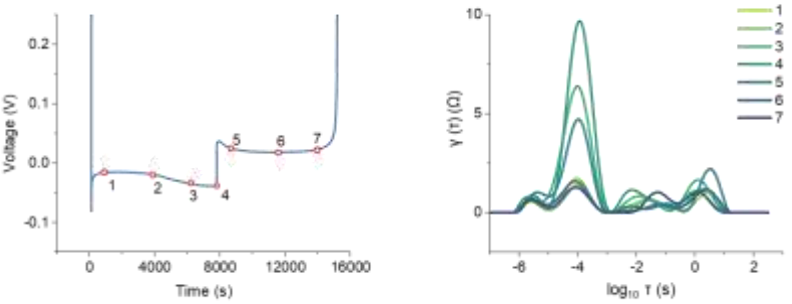


Supplementary Figure 19. Voltage profile with noted time for EIS acquisition and corresponding DRT analysis during lithium plating/stripping.

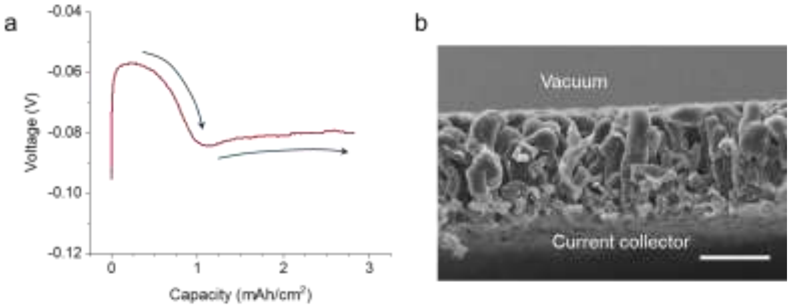


Supplementary Figure 20. (**a**) Discharge voltage profile with increasing lithium deposition capacity at 0.5 mA cm$^{-2}$. (**b**) Post-deposition cross-sectional SEM images. Scale bar 10 μm.

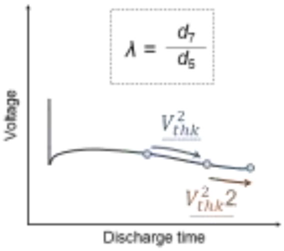


Supplementary Figure 21. Schematic for the definition of the composite feature λ, representing the structural characteristics of lithium deposits.

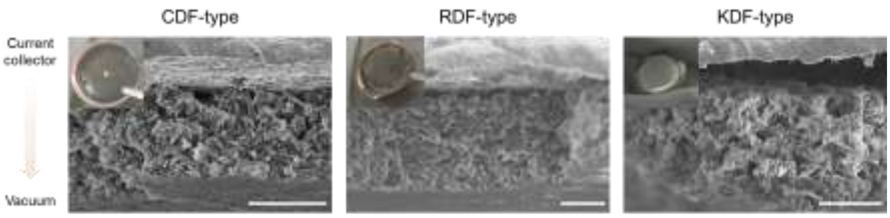


Supplementary Figure 22. Cross-sectional SEM images of initial lithium deposits for each failure type at 0.5 mA cm$^{-2}$ and 1 mAh cm$^{-2}$, along with their optical photographs. Scale bars 10 μm.

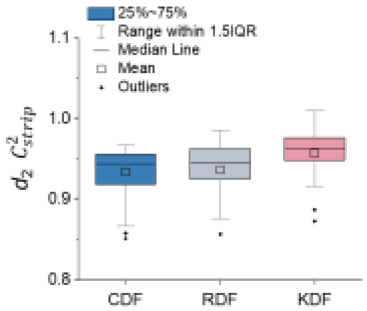


Supplementary Figure 23. Distribution analysis of CE ($d_2$, $C^2_{strip}$) for lithium metal anodes across failure types.

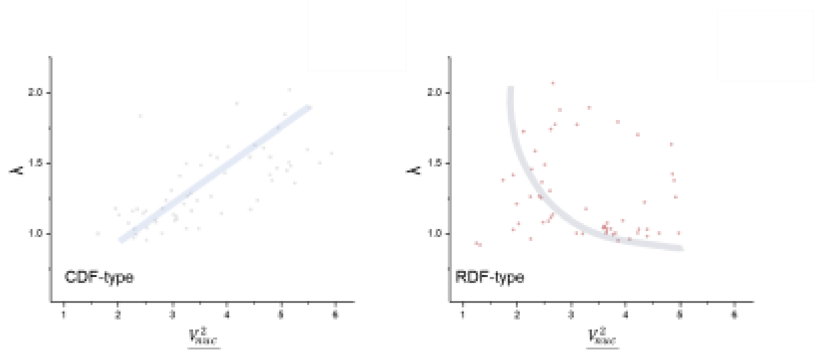


Supplementary Figure 24. Correlation between microstructure and nucleation-related features for lithium metal anode for different failure types.

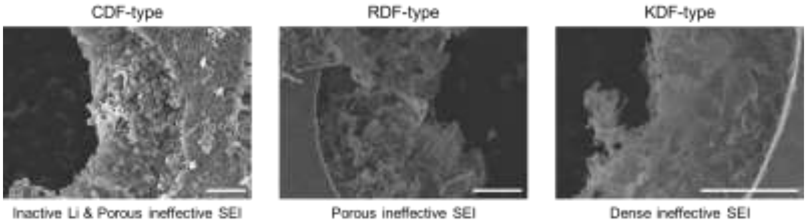


Supplementary Figure 25. SEM images of post-cycling lithium metal anodes for each failure type. Scale bars 10 μm.

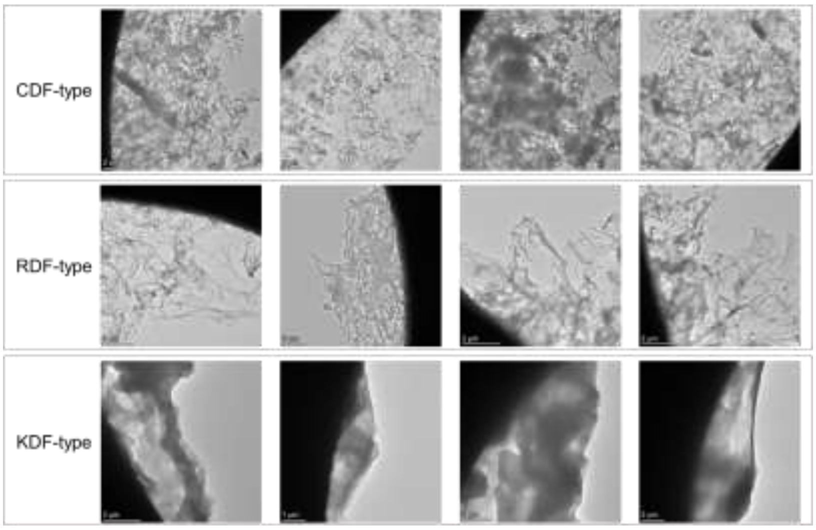


Supplementary Figure 26. TEM images of post-cycling lithium metal anodes from various locations for each failure type. Scale bars 2 μm.

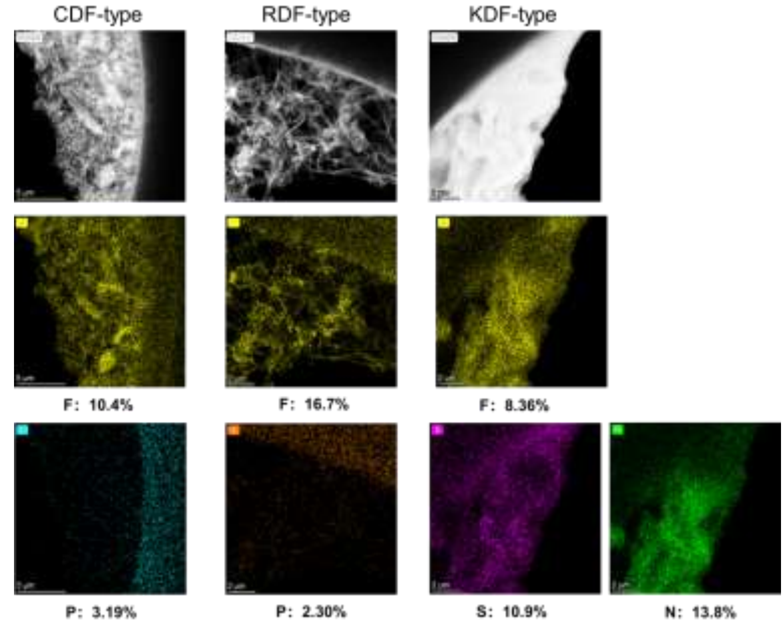


Supplementary Figure 27. Distribution of key elements identifying inorganic components in the SEI for different failure types. Scale bars 2 μm.

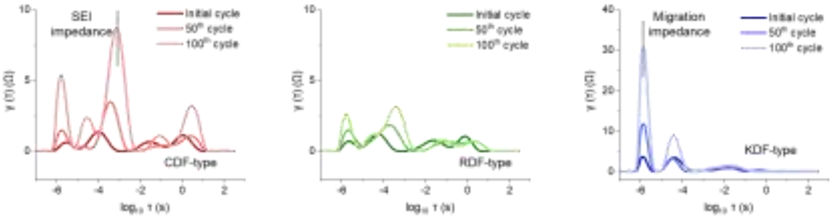


Supplementary Figure 28. DRT analysis of EIS results over cycles for each failure type.

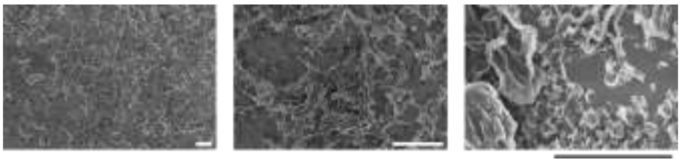

Supplementary Figure 29. SEM images of ineffective SEI formation after one cycle of lithium plating/stripping. Scale bars 10 μm.

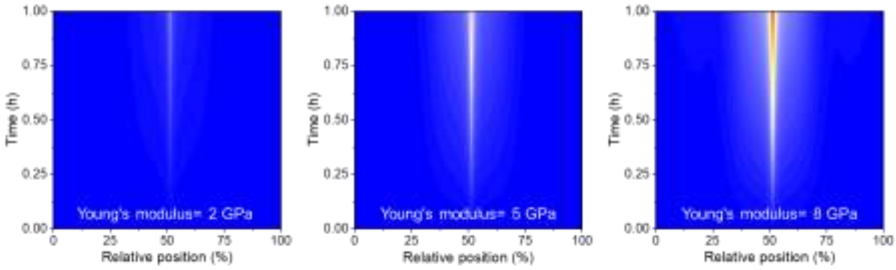


Supplementary Figure 30. Simulation of stress distribution during lithium plating with SEIs with varying Young's modulus.

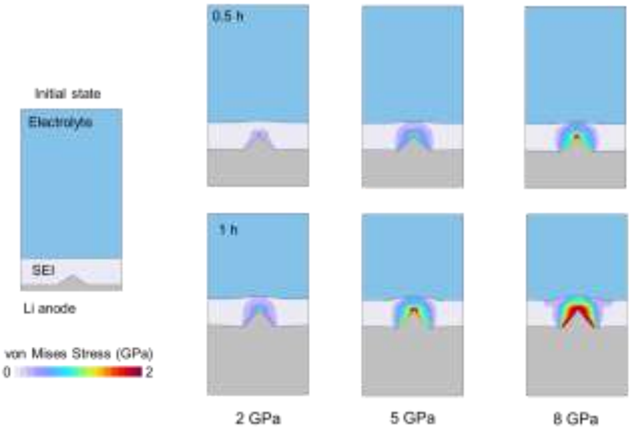


Supplementary Figure 31. Visual display of stress distribution during lithium plating with SEIs with varying Young's modulus.

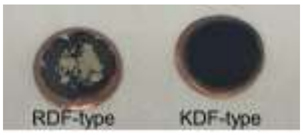


Supplementary Figure 32. Optical photographs of lithium metal anode post-cycling for different failure types.

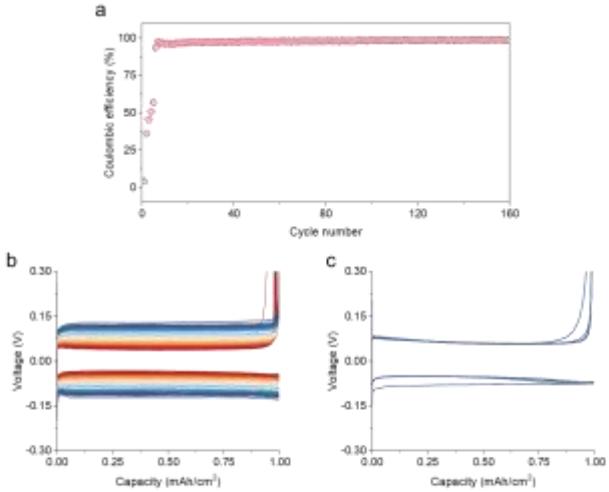


Supplementary Figure 33. (**a and b**) Cycling performance of lithium metal anode for a KDF-type cell and its voltage evolution profile. (**c**) Voltage profile of lithium half-cells using the degraded electrolyte.

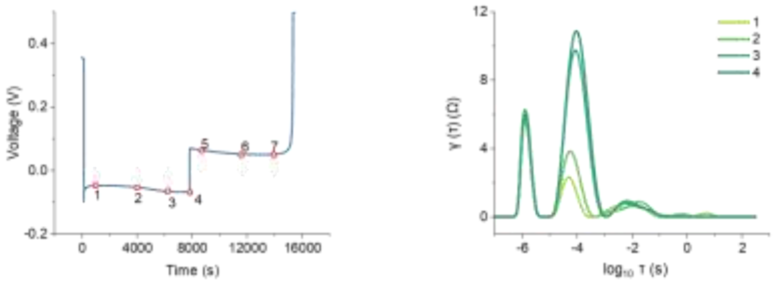


Supplementary Figure 34. Voltage profile with noted time for EIS acquisition using the degraded electrolyte and DRT analysis for obtained EIS.

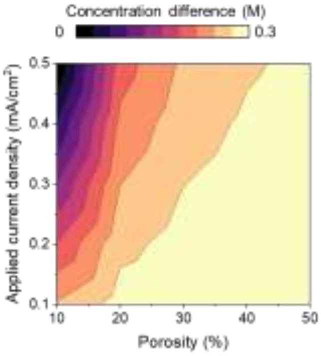


Supplementary Figure 35. Simulation of concentration difference within the ineffective SEI with different porosity at different current densities assuming electrolyte concentration is 1 M.

## Supplementary Tables

**Supplementary Table 1** Feature engineering.

| Feature number | Feature name | Feature meaning | Feature calculation |
|---|---|---|---|
| 1 | $V_{nuc}^{1}$ | First cycle lithium nucleation overpotential | Minimum discharge voltage at normalized discharge capacity between 0 and 0.1 |
| 2 | $\underline{V_{nuc}^{1}}$ | Relative overpotential of the first cycle lithium nucleation compared to the lithium growth plateau overpotential | Feature 1/Feature 4 |
| 3 | $C_{nuc}^{1}$ | First cycle lithium nucleation capacity | Normalized discharge capacity corresponding to discharge voltage of [Feature 4 * 1.5] |
| 4 | $V_{grth}^{1}$ | First cycle lithium growth plateau overpotential | Maximum discharge voltage at normalized discharge capacity between 0.1 and 1 |
| 5 | $V_{grth}^{1}2$ | First cycle lithium deposition end overpotential | Discharge voltage at normalized discharge capacity of 1 |
| 6 | $C_{grth}^{1}$ | Capacity corresponding to the minimum overpotential of the first cycle lithium growth plateau | Normalized discharge capacity corresponding to discharge voltage of [Feature 4] |
| 7 | $V_{strip}^{1}$ | First cycle lithium stripping initial overpotential | Maximum charge voltage at normalized charge capacity between 0 and 0.1 |
| 8 | $V_{strip}^{1}2$ | First cycle lithium stripping plateau overpotential | Minimum charge voltage at normalized charge capacity between 0.1 and 1 |
| 9 | $\underline{V_{strip}^{1}}$ | Relative initial overpotential of the first cycle lithium stripping compared to the lithium stripping plateau overpotential | Feature 7/Feature 8 |
| 10 | $C_{strip}^{1}$ | Capacity corresponding to the end of the first cycle lithium stripping | Normalized charge capacity corresponding to a charge voltage of 0.5 V |
| 11 | $V_{nuc}^{2}$ | Second cycle lithium nucleation overpotential | Minimum discharge voltage at normalized discharge capacity between 0.1 and 1 |
| 12 | $C_{nuc}^{2}$ | Second cycle lithium nucleation capacity | Normalized discharge capacity corresponding to discharge voltage of [Feature 15 * 1.5] |
| 13 | $\underline{V_{nuc}^{2}}$ | Relative overpotential of the second cycle lithium nucleation compared to the lithium growth plateau overpotential | Feature 11/Feature 14 |
| 14 | $V_{grth}^{2}$ | Second cycle lithium growth plateau start overpotential | Discharge voltage at normalized discharge capacity of 0.1 |
| 15 | $V_{grth}^{2}2$ | Second cycle lithium growth plateau overpotential | Minimum discharge voltage at normalized discharge capacity between 0.1 and 0.5 |
| 16 | $V_{grth}^{2}3$ | Second cycle lithium growth plateau end overpotential | Discharge voltage at normalized discharge capacity of 0.5 |
| 17 | $V_{thk}^{2}$ | Second cycle lithium thickening process overpotential | Discharge voltage at normalized discharge capacity of 0.8 |
| 18 | $V_{thk}^{2}2$ | Second cycle lithium deposition end overpotential | Discharge voltage at normalized discharge capacity of 1 |
| 19 | $\underline{V_{thk}^{2}}$ | Overpotential change from the end of the second cycle lithium growth plateau to the start of thickening | Feature 17/Feature 16 |
| 20 | $\underline{V_{thk}^{2}}2$ | Overpotential change from the start of the second cycle lithium thickening to the end of deposition | Feature 18/Feature 17 |
| 21 | $V_{strip}^{2}$ | Second cycle lithium stripping initial overpotential | Maximum charge voltage at normalized charge capacity between 0 and 0.1 |
| 22 | $V_{strip}^{2}2$ | Second cycle lithium stripping plateau overpotential | Minimum charge voltage at normalized charge capacity between 0.1 and 1 |
| 23 | $\underline{V_{strip}^{2}}$ | Relative initial overpotential of the second cycle lithium stripping compared to the lithium stripping plateau overpotential | Feature 21/Feature 22 |

| | | | |
|---|---|---|---|
| 24 | $C_{strip}^{2}$ | Capacity corresponding to the end of the second cycle lithium stripping | Normalized charge capacity corresponding to a charge voltage of 0.5 V |

Underlined features representing relative values and non-underlined features indicating absolute values.

**Supplementary Table 2** Time required to determine the failure type (curve evolution) for each sample.

| Method | Base time cost | | | | Boundary time cost of each sample | | | | | |
|---|---|---|---|---|---|---|---|---|---|---|
| Traditional post-mortem method | / | | | | Cell assembly/h | Rest/h | | Long-term cycling/h | | Sum/h |
| | | | | | 0.08 | 10.00 | | 456.00 | | 466.08 |
| Our pre-mortem prediction method | Model construction | | | Sum/h | Model use | | | | | Sum/h |
| | Database construction/h | Feature engineering/h | Model training and testing/h | | Cell assembly/h | Rest/h | Initial cycling/h | Data format conversion/h | ML prediction/h | |
| | 24.00 | 72.00 | 0.43 | 96.43 | 0.08 | 10.00 | 8.00 | 0.02 | 0.00 | 18.10 |

**Supplementary Table 3.** Samples for characterizations and validations.

| Sample number | Electrolyte composition | Reference | Use |
|---|---|---|---|
| 1 | 1 M $LiPF_6$ in EC:EMC (1:1 v/v) + 2% FEC | [4] | Major characterizations |
| 2 | 1 M $LiPF_6$+0.1 M LiDFOB in FEC:FEMC (1:3 v/v) | [5] | Major characterizations |
| 3 | 1.5 M LiFSI in DMOTFS | [6] | Major characterizations |
| 4 | 1 M $LiPF_6$ in EC:EMC:DMC (1:1:1 v/v/v) | [7] | Validation of ML model (Figure S8a, Figure 2h) |
| 5 | 1 M LiTFSI in EC:DEC (1:1 v/v) | [8] | Validation of ML model (Figure S8b, Figure 2h) |
| 6 | LiFSI:DME:TTE, 1:1.2:3 (m/m/m) | [9] | Validation of ML model (Figure S8c, Figure 2h) |
| 7 | 1 M $LiPF_6$ in DMC:FEC (8:2 v/v) + 1% SN | [10] | Validation of ML model (Figure S8d, Figure 2h) |
| 8 | 2.1 M LiFSI in DMES | [11] | Validation of ML model (Figure S8e, Figure 2h) |
| 9 | 1 M $LiPF_6$ in EC:DMC (1:1 v/v) | [11] | SEI composition and modulus validation (Figure S11b, S12a, S15a)<br>Validation of ML model (Figure S8f, Figure 2h) |
| 10 | 1 M LiTFSI in DME:DOL (1:1 v/v) + 5% $LiNO_3$ | [12] | SEI composition validation (Figure S11c, S12b)<br>Validation of ML model (Figure S8g, Figure 2h) |
| 11 | LiFSI:DME:TTE, 1:9:27 (m/m/m) | [9] | SEI composition validation (Figure S11d, S12c)<br>Validation of ML model (Figure S8h, Figure 2h) |
| 12 | 1 M $LiPF_6$ in DMC:FEC (8:2 w/w) | [13] | SEI modulus validation (Figure S15b)<br>Validation of<br>.0 |

| | | | |
|---|---|---|---|
| | | | .0el (Figure S8i, Figure 2h) |
| 13 | 1.5 M LiFSI in DMMS | [11] | SEI composition and modulus validation<br>(Figure S15c, S16)<br>Validation of ML model (Figure S8j, Figure 2h) |

## Supplementary References

1. Wan TH, Saccoccio M, Chen C *et al.* Influence of the discretization methods on the distribution of relaxation times deconvolution: Implementing radial basis functions with DRTtools. *Electrochim Acta* 2015; **184**: 483-99.
2. Fang C, Li J, Zhang M *et al.* Quantifying inactive lithium in lithium metal batteries. *Nature* 2019; **572**: 511-5.
3. He Y, Jiang L, Chen T *et al.* Progressive growth of the solid-electrolyte interphase towards the Si anode interior causes capacity fading. *Nat Nanotechnol* 2021; **16**: 1113-20.
4. Piao Z, Xiao P, Luo R *et al.* Constructing a stable interface layer by tailoring solvation chemistry in carbonate electrolytes for high-performance lithium-metal batteries. *Adv Mater* 2022; **34**: e2108400.
5. Piao Z, Ren HR, Lu G *et al.* Stable operation of lithium metal batteries with aggressive cathode chemistries at 4.9 V. *Angew Chem Int Ed* 2023; **62**: e202300966.
6. Piao Z, Wu X, Ren HR *et al.* A semisolvated sole-solvent electrolyte for high-voltage lithium metal batteries. *J Am Chem Soc* 2023; **145**: 24260-71.
7. Liu W, Li J, Li W *et al.* Inhibition of transition metals dissolution in cobalt-free cathode with ultrathin robust interphase in concentrated electrolyte. *Nat Commun* 2020; **11**: 3629.
8. Moon J, Kim DO, Bekaert L *et al.* Non-fluorinated non-solvating cosolvent enabling superior performance of lithium metal negative electrode battery. *Nat Commun* 2022; **13**: 4538.
9. Cui Z, Jia Z, Ruan D *et al.* Molecular anchoring of free solvents for high-voltage and high-safety lithium metal batteries. *Nat Commun* 2024; **15**: 2033.
10. Zhang Y, Cao Y, Zhang B *et al.* Rational molecular engineering via electron reconfiguration toward robust dual-electrode/electrolyte interphases for high-performance lithium metal batteries. *ACS Nano* 2024; **18**: 14764-78.
11. Huang Y, Li R, Weng S *et al.* Eco-friendly electrolytes via a robust bond design for high-energy Li metal batteries. *Energy & Environ Sci* 2022; **15**: 4349-61.
12. Gao R, Zhang M, Han Z *et al.* Unraveling the coupling effect between cathode and anode toward practical lithium-sulfur batteries. *Adv Mater* 2024; **36**: e2303610.
13. Fan X, Chen L, Borodin O *et al.* Non-flammable electrolyte enables Li-metal batteries with aggressive cathode chemistries. *Nat Nanotechnol* 2018; **13**: 715-22.
